# Neural stem cell-derived small extracellular vesicles: a new therapy approach in neurological diseases

**DOI:** 10.3389/fimmu.2025.1548206

**Published:** 2025-04-16

**Authors:** Mengyao Wang, Dongdong Chen, Renjie Pan, Yue Sun, Xinyu He, Youming Qiu, Yuexin Hu, Xiangsheng Wu, Xuxiang Xi, Rong Hu, Zhigang Jiao

**Affiliations:** ^1^ Department of Laboratory Medicine, First Affiliated Hospital of Gannan Medical University, Ganzhou, China; ^2^ College of Medical Technology, Gannan Medical University, Ganzhou, China; ^3^ The First School of Clinical Medicine, Gannan Medical University, Ganzhou, China; ^4^ Precision Medicine Center, First Affiliated Hospital of Gannan Medical University, Ganzhou, Jiangxi, China; ^5^ Key Laboratory of Prevention and Treatment of Cardiovascular and Cerebrovascular Diseases, Ministry of Education, Gannan Medical University, Ganzhou, Jiangxi, China

**Keywords:** neural stem cell, small extracellular vesicles, NSC-derived small extracellular vesicles, neurological diseases, neuroprotective

## Abstract

Neural stem cells (NSCs) possess pluripotent characteristics, proliferative capacity, and the ability to self-renew. In the context of neurological diseases, transplantation of NSCs has been shown to facilitate neurological repair through paracrine mechanisms. NSC-derived small extracellular vesicles (NSC-sEVs), a prominent component of the NSC secretome, play a crucial role in modulating various physiological and pathological processes, such as regulating the NSC microenvironment, promoting endogenous NSC differentiation, and facilitating the maturation of neurons and glial cells. Moreover, NSC-sEVs exhibit reduced immunogenicity, decreased tumorigenic potential, and enhanced ability to traverse the blood-brain barrier. Consequently, NSC-sEVs present novel therapeutic approaches as non-cellular treatments for neurological disorders and are poised to serve as a viable alternative to stem cell therapies. Furthermore, NSC-sEVs can be manipulated to enhance production efficiency, improve biological activity, and optimize targeting specificity, thereby significantly advancing the utilization of NSC-sEVs in clinical settings for neurological conditions. This review provides a comprehensive overview of the biological functions of NSC-sEVs, their therapeutic implications and underlying molecular mechanisms in diverse neurological disorders, as well as the potential for engineering NSC-sEVs as drug delivery platforms. Additionally, the limitations and challenges faced by NSC-sEVs in practical applications were discussed in depth, and targeted solutions were proposed.

## Background

Neurological disorders represent a significant burden on global public health, being a prominent contributor to disability and mortality (1). The prevalence of neurological disorders is expected to rise annually due to the aging population (2). A primary characteristic of neurological diseases is the gradual deterioration of neurons (3, 4). There is currently no clinical cure for any of the various neurological disorders, as the regeneration of neurons pose a significant challenge; therefore, current interventions focus on symptom management and disease progression mitigation (5). In recent years, the transplantation of stem cells has been investigated as a potential therapeutic approach for a range of neurological disorders.

Neural stem cells (NSCs) are of particular interest due to their unique characteristics as self-renewing, multipotent progenitors capable of differentiating into neurons and various types of glial cells, such as astrocytes and oligodendrocytes. Certain marker proteins, including Pax6, Sox2, Olig2, Blbp, and Nestin, serve as distinguishing features of neural stem cells (NSCs) in comparison to other cell types (6). Anatomical and molecular biological studies have revealed that endogenous NSCs in adults are primarily situated in the subgranular zone of the hippocampal dentate gyrus and the subventricular zone of the forebrain (7). Endogenous NSCs typically remain quiescent, yet can be induced to proliferate and differentiate into neural cells that migrate to areas of tissue damage for repair when stimulated by external factors (8). Extensive research conducted in recent decades has demonstrated the significant contribution of NSCs to neurogenesis and tissue repair within the central nervous system, leading to the development of NSCs transplantation as a promising therapeutic approach for various neurological disorders (9). In terms of the efficacy of NSCs transplantation, it has been demonstrated to ameliorate dyskinesia in individuals with Parkinson’s disease (PD) (10), alleviate spasticity and nerve damage in stroke patients (11), enhance cognitive function in an Alzheimer’s disease (AD) model (12) and promote brain repair following a stroke (13). The therapeutic mechanism of NSCs therapy primarily involves proliferation, differentiation, and paracrine signaling. For instance, transplanted NSCs have the capacity to undergo differentiation into neurons for the purpose of replacing damaged neurons, or alternatively, to secrete cytokines and neurotrophic factors that facilitate neurogenesis and angiogenesis through paracrine signaling. This process serves to mitigate neuroinflammation and ultimately promote the restoration of the nervous system (14–17). Despite the considerable potential of NSCs therapy in the treatment of neurological disorders, challenges such as immunosuppressive rejection, tumorigenic risks, source availability, and delivery efficacy remain significant limitations (18). Consequently, stem cell replacement therapy has emerged as a subject of heightened interest within the academic community. Recent research has indicated that the therapeutic properties of NSCs are primarily facilitated by their secretome, comprising neurotrophic factors, microRNAs, proteins, and small extracellular vesicles (sEVs) (19). These sEVs, a crucial constituent of the secretome of NSCs, play essential roles in intercellular signaling and material transfer mechanisms (20). Furthermore, as membrane-bound vesicles released by NSCs, sEVs encapsulate cytokines, growth factors, genetic material, and other bioactive molecules from the originating cells, enabling the transfer of biologically active constituents and overcoming biological barriers. Therefore, sEVs originating from NSCs are deemed suitable for application in stem cell replacement therapies (21, 22).

## sEVs

In earlier scientific literature, sEVs were commonly referred to as “exosomes,” a term originating from their initial discovery in reticulocytes and their early characterization as cellular byproducts (23). These nanoscale lipid bilayer vesicles, with diameters ranging from 40 to 160 nm, are composed of proteins, nucleic acids, lipids, and metabolites (24). Subsequent research has demonstrated that sEVs contain a variety of bioactive molecules that play a crucial role in mediating intercellular communication (25). However, in accordance with the updated guidelines of MISEV2018, the term “sEVs” is now preferred to describe these vesicles, as it avoids the historical ambiguities and conflicting definitions associated with the term “exosome,” as well as unrealistic expectations regarding their unique biogenesis (26). Therefore, this review will consistently employ the term “sEVs” to refer to these small extracellular vesicles. The secretion of sEVs is a common phenomenon among most cells, with these vesicles being found in a variety of bodily fluids such as blood, urine, saliva, amniotic fluid, breast milk, and cerebrospinal fluid. However, it is important to note that sEVs derived from different sources exhibit variations in their composition and biological functions (27, 28). Within the nervous system, NSC-sEVs play a crucial role in modulating processes such as cell proliferation, neurogenesis, synaptic plasticity, neuroinflammation, neuroprotection, vascular endothelial cell generation, and the maintenance of central nervous system homeostasis (29–31). Furthermore, NSC-sEVs exhibit low immunogenicity, favorable biocompatibility, and efficient blood-brain barrier traversal capacity. Given these characteristics, NSC-sEVs hold great promise as therapeutic agents for neurological diseases.

## Biogenesis and composition of sEVs

The precise mechanism of sEVs formation remains incompletely understood, with current research focusing on the endosomal sorting complex required for transport (ESCRT) dependent and ESCRT-independent pathways. The ESCRT-dependent pathway, involving ESCRT-0 complex (containing hepatocyte growth factor-regulated tyrosine kinase substrate (HRS) and signal transducing adaptor molecule, which form a 1:1 complex in solution), ESCRT-I (a heterotetrameric complex with an elongated shape, consisting of the core components tumor susceptibility gene 101 protein, vacuolar protein sorting 28, vacuolar protein sorting and multivesicular bodies 12 or Ubiquitin Associated Protein 1), ESCRT-II complex (a heterotetramer of three winged-helix domain containing proteins, containing vacuolar protein sorting 36, vacuolar protein sorting 22, vacuolar protein sorting 25, with the latter occurring in two copies), ESCRT- III (a biochemically less defined complex, consists of oligomers or polymers of small α-helical charged multivesicular body protein), represents the classical pathway of sEVs formation (32–35). The ESCRT machinery functions in a sequential manner to facilitate the recognition, aggregation, deformation, and separation of ubiquitinated proteins on endosomal membranes. Specifically, ESCRT-0 recognizes ubiquitinated proteins and aggregates them on endosomal membranes through interactions with HRS lattice proteins (36); while ESCRT-I and ESCRT-II work collaboratively to deform and bend the endosomal membranes to form buds (37); ESCRT-III then cleaves the membrane neck from the inside to drive vesicle separation (38). Ultimately, the multivesicular body (MVB) can undergo degradation by lysosomes or fusion with the plasma membrane, leading to the release of intraluminal vesicles (ILVs) outside the cell to form sEVs. Relatively little research has been carried out on the ESCRT-independent pathway. Candia et al. showed that Rab31 is recruited into ceramide- and cholesterol-containing membranes by interacting with flotillin to regulate small extracellular vesicle biogenesis by stimulating ILV outgrowth and EGFR packaging in an ESCRT-independent pathway (39). On the other hand, Rab31 inhibits the fusion of MVB with lysosomes via RAB7, thereby preventing EGFR lysosomal degradation and allowing ILV to be secreted into small extracellular vesicles (40). RAB31 promotes sEVs biogenesis through dual action in an ESCRT-independent pathway, and the RAB31-FLOTs mechanism plays an important role in the secretory synthesis of small extracellular vesicles.

**Figure 1:**
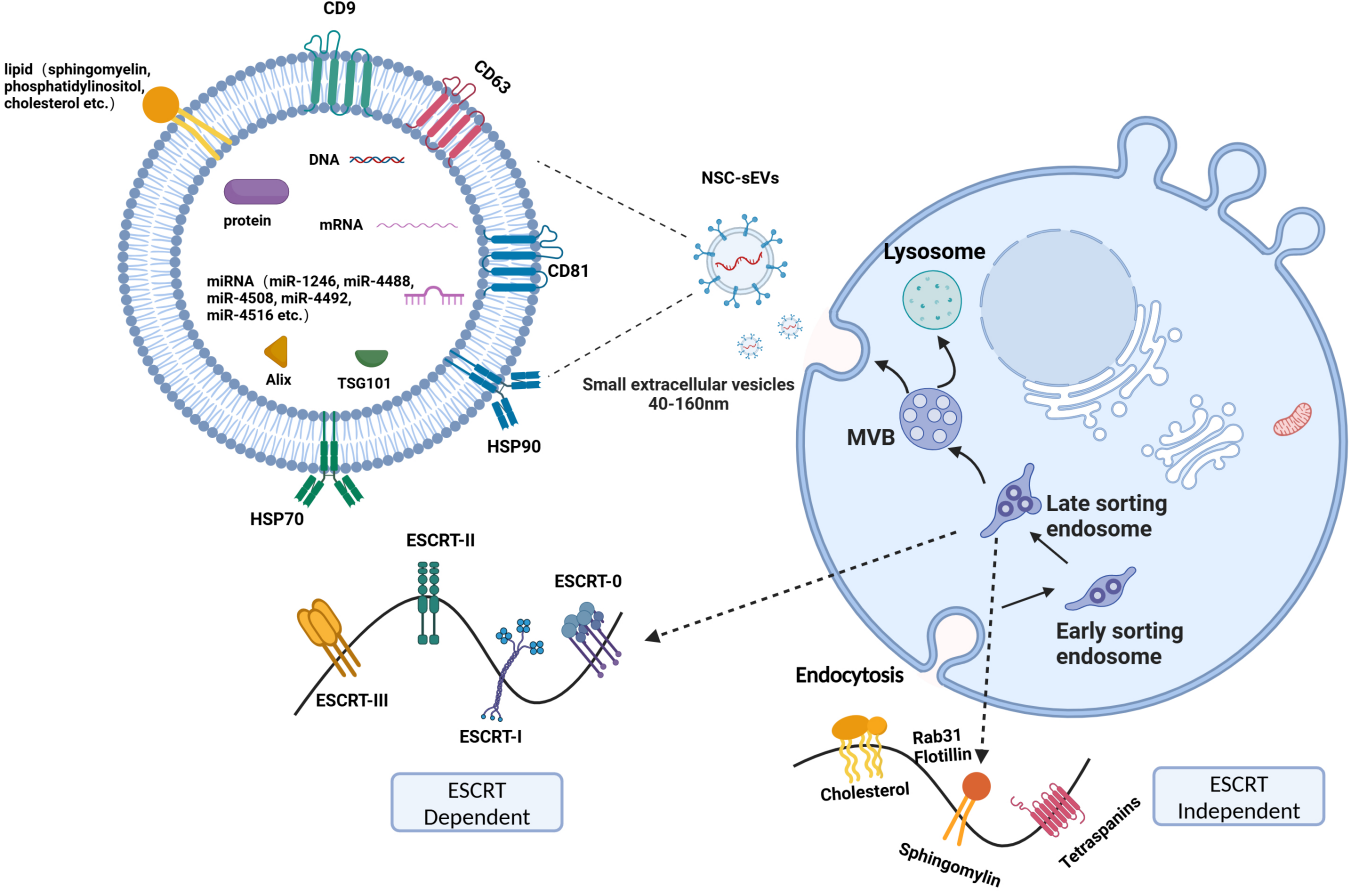
ESCRT dependent and ESCRT-independent pathways. The process of sEVs production mainly involves the invagination of the cell membrane to form endosomes, the formation of intracellular multivesicular bodies (MVBs) by secondary invagination of endosomes, and finally the fusion of MVBs with the cytoplasmic membrane to secrete intraluminal vesicles (ILVs) outside the cell to form sEVs. The primary constituents of sEVs include proteins, nucleic acids, and lipids.

Studies conducted by Exocarta (http://www.exocarta.org) revealed the presence of 9769 proteins, 3408 mRNAs, and 2838 miRNAs within sEVs from more than 286 sEVs studies (**Figure 1**). In addition, various lipids, such as sphingomyelin, phosphatidylserine, phosphatidylcholine, phosphatidylinositol, ceramide, and cholesterol, are also present in sEVs. Notably, sEVs from various sources exhibit common protein markers, such as CD9, CD63, CD81, tumor susceptibility gene 101 protein, ALG-2 interacting protein X, and heat shock proteins (heat shock protein 70 and heat shock protein 90), which help differentiate them from other types of extracellular vesicles (41). The composition of sEVs, particularly in terms of proteins and miRNAs, can vary significantly due to their diverse sources (42–45). Even the same protein in sEVs from diverse sources exerted significantly different roles *in vitro* and *in vivo*. For example, the upregulation of protein pentraxin 3 (PTX3) within NSC-sEVs derived from human-induced pluripotent stem cells attenuated the inflammatory response induced by activated human microglia (46). In another study, induction of PTX3 upregulation in induced vascular progenitor cell (iVPC)-derived sEVs promoted angiogenesis and improved microcirculation in a rat hindlimb ischemia model (47). Conversely, PTX3 present in sEVs from breast cancer cells subjected to chemotherapy facilitated pulmonary metastasis by priming the premetastatic niche (48). In conclusion, the roles of identical substances within small extracellular vesicles (sEVs) secreted by different cell types seem to exhibit significant variability. Furthermore, the functions of the abundant substances contained within sEVs appear to be intricately linked to their cellular origin of secretion.

## NSC-sEVs and MSC-sEVs

sEVs are increasingly recognized for their potential therapeutic applications in various neurological disorders. Notably, NSC-sEVs and mesenchymal stem cell-derived small extracellular vesicles (MSC-sEVs) have emerged as significant focal points in contemporary research. NSCs are predominantly located in the central nervous system, whereas mesenchymal stem cells (MSCs) are broadly distributed across various tissues, including bone marrow, umbilical cord, adipose tissue, and dental pulp (49). Despite the similarities between NSCs and MSCs in terms of self-renewal, multipotent differentiation, immunomodulation, and tissue repair capabilities, MSCs are primarily characterized by specific biomarkers such as CD29 and CD166 (50). However, NSCs possess distinct advantages in the context of neurological disease treatment. They have the capacity to directly differentiate into various neural cell types, thereby replacing damaged neurons and glial cells and facilitating the repair of neural tissue. Additionally, NSCs can activate endogenous repair mechanisms through the secretion of neurotrophic factors. In contrast, MSCs are capable of differentiating into neural-like cells only under specific conditions. MSCs have demonstrated significant efficacy in alleviating neuroinflammation and promoting the recovery of neural function (51, 52). Nevertheless, differences in the transcriptional profiles between NSCs and MSCs have been identified (53). These variations result in differences in the cargo of sEVs, which may contribute to therapeutic failures in the context of neurological disease treatment. Notably, the neuroprotective effects of MSCs are largely mediated through their paracrine release of small molecules and sEVs, a mechanism that aligns with the pathway through which NSCs exert their protective effects.

Compared to MSC-sEVs, NSC-sEVs present several notable advantages for neurological applications. Primarily, NSC-sEVs are derived from neural stem cells, which possess the capacity to differentiate into neurons, glial cells, and oligodendrocytes. This characteristic potentially endows NSC-sEVs with a higher concentration of bioactive molecules directly pertinent to neuronal function, including brain-derived neurotrophic factor (BDNF), glial-derived neurotrophic factor (GDNF), nerve growth factor (NGF), among others. These factors are instrumental in promoting neuronal survival, differentiation, and axonal growth (14, 16, 54). Furthermore, NSC-sEVs contain a variety of miRNAs (miR-138-5p, hsa-miR-206, hsa-mir-182-5p, hsa-miR-133a-3p and hsa-miR-3656) that regulate neurogenesis, synaptic plasticity, and neuroprotective functions (55–57). These components collectively form the foundation of their unique neural regulatory capabilities. In contrast, although MSC-sEVs also contain certain active factors that promote tissue repair and neural regeneration, the tissue specificity of MSCs, which primarily differentiate into bone, cartilage, and fat cells, limits the direct role of MSC - sEVs to some extent in the restoration of neuronal function (58). The primary role of MSC-sEVs lies in offering a conducive microenvironment for neural regeneration, demonstrating broader regenerative properties. However, they may not be as specifically tailored to the unique requirements of the nervous system as NSC-sEVs (59–61). Therefore, in the context of treating and repairing neurological disorders, NSC-sEVs may demonstrate superior therapeutic potential and efficacy due to their enhanced neuronal relevance and specificity.

Additionally, NSC-sEVs exhibit significant advantages in targeting neuronal cells and uptake efficiency when compared to MSC-sEVs. Proteins such as L1 cell adhesion molecule (L1CAM) and polysialylated neuronal cell adhesion molecule present on the surface of NSC-sEVs facilitate their binding to and endocytosis by neurons and glial cells more effectively (62–64). The optimized tropism for targeting the central nervous system significantly augments their capacity to serve as therapeutic carriers, facilitating the precise delivery of therapeutic agents to specific neuronal cells, which is crucial for improving therapeutic efficacy. Conversely, while MSC-sEVs are widely available and capable of functioning across various tissues, their targeting efficiency towards neuronal cells is relatively limited, resulting in a more diffuse distribution (65). Although this property presents a broad spectrum of potential applications for MSC-sEVs in the treatment of various diseases, NSC-sEVs exhibit superior efficacy in therapeutic contexts that necessitate a heightened focus on the nervous system.

Research has demonstrated that NSC-sEVs exhibit superior efficacy in animal models of neurological disorders when compared to MSC-sEVs. Specifically, study compared the physical characteristics and biological functions of NSC-sEVs and MSC-sEVs. Although NSC-sEVs and MSC-sEVs are no significant difference on the physical characteristics and biodistribution, NSC-sEVs significantly outperformed MSC-sEVs in the murine model of stroke (66). This superiority was evidenced by a more substantial reduction in injury volume, enhanced functional recovery, and a more precise modulation of the inflammatory response in the mice. Subsequent research has demonstrated that NSC-sEVs significantly enhance neuronal survival, axonal regeneration, and synaptic plasticity. These mechanisms synergistically contribute to the repair and reconstruction of the nervous system, thereby establishing a robust foundation for the extensive application of NSC-sEVs in the treatment of neurodegenerative diseases (66). The augmented therapeutic efficacy of NSC-sEVs is likely due to their superior capacity to emulate the natural paracrine signaling of neural stem cells. NSC-sEVs have demonstrated superior efficacy in stimulating endogenous repair mechanisms and enhancing neuroplasticity in injured brain tissue. This includes promoting the proliferation and differentiation of resident neural progenitor cells and modulating inflammatory responses, thereby fostering an environment more conducive to neural repair. Existing research indicates that NSC-sEVs possess distinct advantages over MSC-sEVs in the treatment of neurological disorders. The unique molecular cargo and optimized targeting capabilities of NSC-sEVs render them a promising therapeutic strategy. Consequently, this study will concentrate on NSC-sEVs in the subsequent sections.

## Biological functions of NSC-sEVs

**Figure 2:**
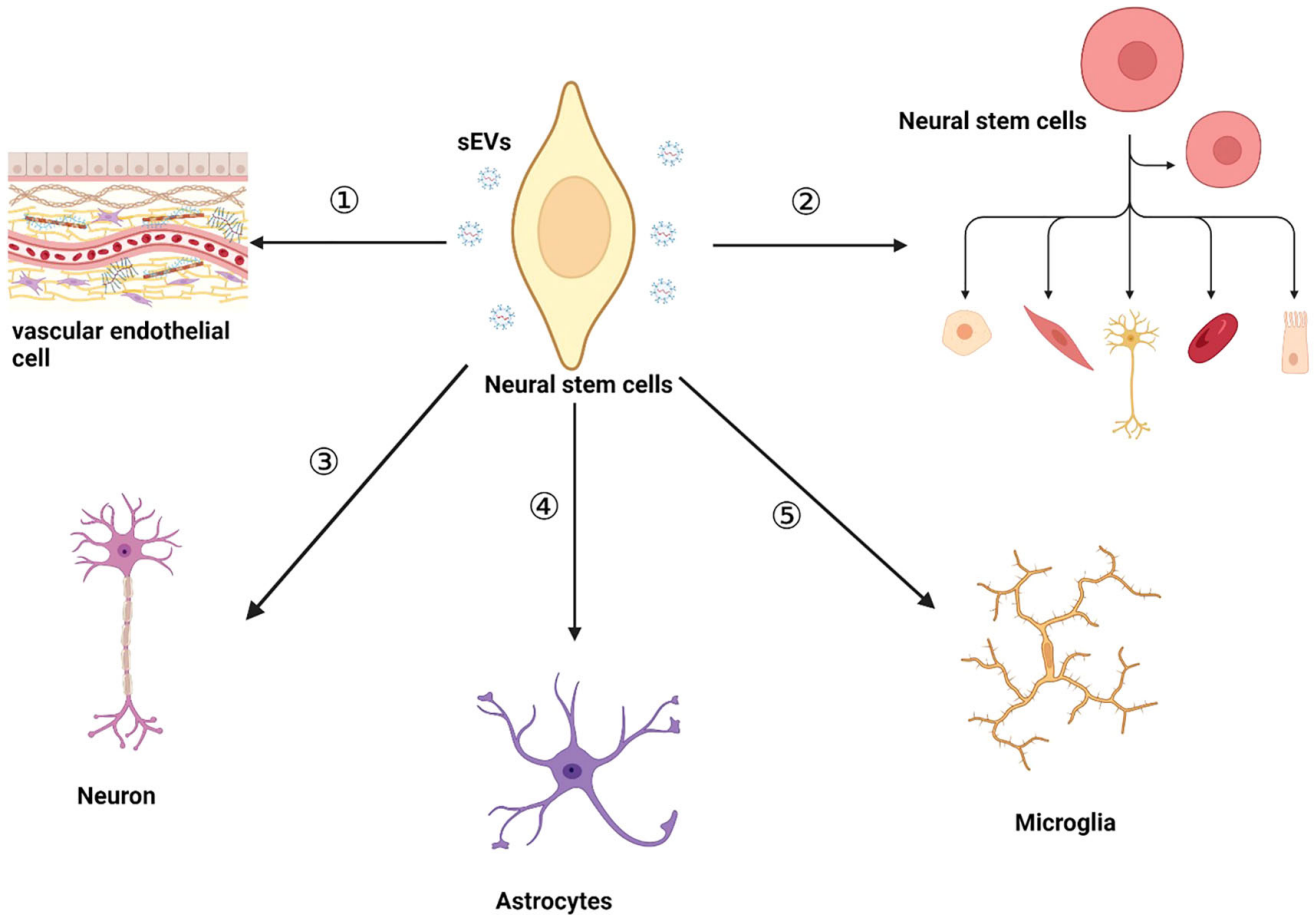
The biological functions of NSC-sEVs within the nervous system include:① NSC-sEVs maintain cellular homeostasis or modulate the extracellular milieu for intercellular communication. ② NSC-sEVs promote the proliferation and differentiation of endogenous NSC and maintain endogenous NSC quiescence. ③ NSC-sEVs inhibit neuronal apoptosis and have a protective effect on neurons. ④NSC-sEVs have a significant protective effect on astrocytes suffering from ischemic injury. ⑤ NSC-sEVs regulate the cellular morphology of microglia and inhibit the hyperactivation of microglia.

An increasing amount of research indicates that NSC-sEVs play a neuroprotective role in the neurodegeneration. For example, analysis of NSC-sEVs using small RNA sequencing, proteomics, and pathway analysis has shown that NSC-sEVs are enriched in miRNAs and proteins associated with neuroprotective, anti-apoptotic, inflammation-suppressing, anti-oxidative stress, and Aβ (amyloid-β protein)-reducing activities (44). Notably, highly expressed miRNAs, such as miR-1246, miR-4488, miR-4508, miR-4492, miR-4516, miR-320a, miR-320b, miR-103a-3p, miR-21-5p, miR-26a-5p, miR-30a-3p, miR-181a-5p, miR-191-5p have been identified in NSC-sEVs (44, 67). These miRNAs play crucial roles in diverse cellular signaling pathways. For instance, the highly expressed miR-21-5p in NSC-sEVs can downregulate the mRNA and protein expression levels of TNF-α, promoting the transformation of pro-inflammatory microglia into a non-inflammatory phenotype, thereby playing a crucial anti-inflammatory regulatory role in the nervous system (46). Additionally, Sun Q et al. reported that miR-181a-5p in mouse hippocampal NSCs contributes to neuronal differentiation, thereby alleviating age-related memory impairment and enhancing cognitive abilities (68). Similarly, another study found that direct targeting of MiR-191-5p to DAPK1 reduced Aβ secretion and increased neurite outgrowth in AD, helping to alleviate neuronal cell death (69). A pioneering study by Qi et al. has demonstrated that hippocampal NSC-sEVs are enriched with the long non-coding RNA myocardial infarction associated transcript (MIAT), a gene located at chromosome 22:27,042,392-27,072,441 (GRCh37/hg19). The findings indicate that these sEVs exhibit significant anti-inflammatory and neuroprotective properties in a rat model of vascular dementia. In a subsequent investigation, the administration of sEVs derived from gene-edited NSCs in a rat model of vascular dementia demonstrated that MIAT-containing NSC-sEVs significantly reduced the secretion of inflammatory factors and alleviated oxidative damage. Further investigations revealed that this therapeutic effect was mediated through the modulation of the miR-34b-5p/CALB1 axis by lncRNA MIAT, thereby elucidating the molecular mechanism by which NSC-sEVs attenuate brain inflammation and injury (70). Moreover, Li et al.’s study focused on NSC-sEVs under denervation conditions, in which they found that the expression of 14 circular RNAs was significantly upregulated. Among them, circAcbd6, which acts as an endogenous sponge for miR-320-5p, effectively inhibited the activity of miR-320-5p, which in turn promoted the increased expression of oxysterol-binding protein-related protein 2. This process ultimately promoted NSC differentiation, providing new insights into the potential of NSC-sEVs in the treatment of neurological diseases (71). In summary, the diversity of inclusions in NSC-sEVs may be a key factor for them to show unique efficacy in the treatment of neurological diseases. These findings not only enrich our understanding of the functional complexity of NSC-sEVs, but also open new avenues for the development of novel neuroprotective therapies based on sEVs. In this review, we outline the current biological functions of NSC-sEVs in this context (**Figure 2**). Meanwhile, NSC-sEVs also exerted a regulatory role in neurogenic ecological niches which are consist of the vascular endothelial cell, endogenous NSC, neuronal cells, astrocyte and microglia.

## NSC-sEVs and vascular endothelial cell

Vascular endothelial cells, a type of non-neuronal cell population located within the blood vessels, are integral to the regulation of various physiological processes as a crucial element of the blood-brain barrier and are essential for proper nervous system function (72). Research indicates that NSC-sEVs have the capability to traverse a blood-brain barrier model composed of cerebrovascular endothelial cells, with the internalization of NSC-sEVs by cerebrovascular endothelial cells occurring through heparan sulfate proteoglycans (HSPGs) -dependent endocytosis (30). Meanwhile, NSC-sEVs play a crucial role in preserving the integrity of the blood-brain barrier across various disease models. For instance, Chen et al. demonstrated that NSC-sEVs effectively uphold blood-brain barrier integrity in hypoxic conditions by modulating PTEN expression, enhancing p-Akt expression in cerebrovascular endothelial cells to mitigate apoptosis, and increasing ZO-1 expression, a tight junction-associated protein (73). Similarly, in their study, Zhang and colleagues found that NSC-sEVs attenuated the upregulation of ABCB1 and matrix metalloproteinase 9 expression, as well as inhibited the activation of the NF-κB pathway *in vitro* blood-brain barrier co-culture model. This resulted in a significant enhancement of blood-brain barrier integrity following a stroke event (74). Furthermore, NSC-sEVs notably enhanced the proliferation and migratory capabilities of vascular endothelial cells, thereby facilitating the angiogenesis process (75, 76). Although studies on the mechanisms of how NSC-sEVs maintain the integrity of BBB by regulating vascular endothelial cells are limited, there is little doubt that NSC-sEVs are pivotal in modulating the permeability of the blood-brain barrier in both normal and pathological states. Consequently, targeting NSC-sEVs for drug delivery presents a promising therapeutic approach for addressing blood-brain barrier injury and dysfunction. This strategy holds significant potential and is anticipated to pave the way for novel treatments of neurological disorders.

## NSC-sEVs and endogenous NSC

The NSCs, a population of endogenous stem cells within the central nervous system, is crucial in processes such as neurogenesis, development, and repair (27). NSC-sEVs facilitate intercellular communication, enabling the exchange of genetic information to modulate stemness, self-renewal, and differentiation of stem cells and their subpopulations (77). Emerging research indicates that NSC-sEVs can impact endogenous NSCs proliferation and homeostasis *in vivo* through the transfer of contents such as miRNAs and proteins. For instance, Ma et al. found that the promotion of NSC proliferation by NSC-sEVs is attributed to the activation of the downstream MEK/ERK signaling pathway by growth factor-related proteins (IGF2BP2/3, IGF1/2r, and FGF2) that are abundantly expressed within NSC-sEVs (78). Additionally, it has been demonstrated that NSC-sEVs play a significant role in the differentiation of neural stem cells, as well as in the maturation of neurons and glial cells. Subsequent investigations have identified a high expression of miR-9 within these NSC-sEVs (79). miR-9 demonstrated the ability to specifically target the transcriptional repressor Hes1 and effectively suppress its expression upon internalization by NSCs (79). Nonetheless, prior research has indicated that Hes1, serving as a crucial transcriptional repressor, is essential for the regulation of neural stem cell equilibrium and gliogenesis (80). These studies demonstrate that NSC-sEVs play a crucial role in modulating the differentiation of neural stem cells and the maturation of neurons and glial cells by miR-9-Hes1 axis, offering a novel insight into neurodevelopmental mechanisms. Additionally, Ma et al. also discovered that NPCs (Neural stem/progenitor cells)-derived sEVs exhibit high levels of miR-21a, which enhance NPCs proliferation and neuronal generation (81).

NSC-sEVs not only influence the proliferation and differentiation of NSC but also contribute significantly to the maintenance of NSC homeostasis. For instance, Zhang et al. showed that NSC- sEVs play a role in maintaining the quiescent state of NSC and in the transition between proliferation and quiescence (82). A proteomic analysis comparing proliferating, quiescent, and reactivated NSC-sEVs identified significant differences in protein content and enrichment pathways between quiescent and proliferating sEVs. The findings indicate that NSC-sEVs play a role in regulating NSC quiescence through the modulation of protein synthesis (82). Furthermore, Zhang and colleagues further illustrated that hypothalamic NSC-sEVs harbor distinct microRNAs, including miR-106-5p, miR-20a-5p, and miR-30a-5p, which modulate the aging trajectory by retarding the aging process and prolonging the lifespan of middle-aged mice (83). These investigations highlight the potential of NSC-sEVs to enhance neurogenesis by transferring crucial miRNA and protein cargoes, ultimately contributing to the regeneration of damaged neurons in neurological disorders.

## NSC-sEVs and neuronal cells

Neurons serve as the fundamental units of the nervous system, facilitating the reception, integration, and transmission of information necessary for the regulation of physiological processes and behavioral responses. The presence of dysfunctional neurons is a common feature across a wide range of neurological disorders (84). Therefore, it is imperative to uphold the homeostasis of neuronal function in order to ensure the overall health and functionality of the nervous system. Emerging evidence indicates that NSC-sEVs may exert a neuroprotective effect by modulating various signaling pathways, including anti-oxidative stress, anti-inflammation, and regulation of autophagy flux in dysfunctional or impaired neurons associated with neurodegenerative disorders. For instance, Luo et al. demonstrated that NSC-sEVs reduced the infarction area in a middle cerebral artery occlusion (MCAO) model. Additionally, their research illustrated the significant involvement of miR-150-3p within NSC-sEVs in neuroprotective mechanisms, confirming its role in inhibiting apoptosis of damaged neurons by targeting CASP2 (85).Liu et al. also discovered that NSC-sEVs suppressed oxidative stress and apoptosis by modulating the nuclear translocation of NF-E2-related factor 2 (Nrf2), leading to increased expression of antioxidant enzymes such as superoxide dismutase 1 (SOD1), catalase, and GPx-1 in neurons subjected to hypoxia-reperfusion conditions (86). Qi et al. further illustrated that hippocampal NSC-sEVs mitigated oxidative stress through the upregulation of SOD1 expression, while concurrently downregulating pro-inflammatory factors such as tumor necrosis factor alpha (TNF-α) and interleukin-1 (IL-1) in neurons (70). Moreover, NSC-sEVs safeguard neurons by modulating autophagy flux, thereby eliminating cytoplasmic toxic components and offering protection against neurological diseases (87, 88). For instance, Zhang et al. found that miR-374-5p in NSC-sEVs suppresses neuronal apoptosis by targeting STK-4, thereby promoting autophagic flux and reducing the expression of Bax and caspase-3 in a spinal cord injury model (89). While there is existing literature on the neuroprotective effects and mechanisms of NSC-sEVs on impaired neurons, there is a lack of research on the potential role of NSC-sEVs in the proliferation and maturation of newly generated neurons. Overall, NSC-sEVs have been shown to effectively mitigate neuronal dysfunction through their antioxidant properties, inhibition of apoptosis, and activation of autophagy mechanisms, underscoring their significant contribution to neuroprotection.

## NSC-sEVs and astrocytes

Astrocytes, as one of the glial cell populations, play a key role in the central nervous system (90, 91). The role of neurotoxic reactive astrocytes (A1-astrocytes) in contributing to the progression of neuronal disorders through mechanisms such as glial scarring and chronic neuroinflammation has been well-documented. For instance, A1-astrocytes have been shown to induce cell death in transplanted neural stem cells through the release of complement components and toxic factors, as well as inhibit axon regeneration by promoting the formation of glial scars (92, 93). While some research has explored the effects and underlying mechanisms of modulating astrocyte function using drugs or alternative stem cell types, there is a limited body of literature on the impact of NSC-sEVs on regulating the maturation and function of astrocytes. For example, Zhang and colleagues discovered that NSC-sEVs enhanced the rehabilitation of stroke patients treated with NSCs transplantation by promoting the survival and differentiation of NSCs, as well as the formation of glial scars, through the inhibition of astrocyte activation in a MCAO model (94). Additionally, Sun et al. found that NSC-sEVs exhibited a protective effect on astrocytes against apoptosis, proving to be more effective than induced pluripotent stem cell derived sEVs in the MCAO model (95). However, there is a lack of research on the effector and molecular mechanisms involved in glial cell modulation by NSC-sEVs. It is suggested that the protective abilities of NSC-sEVs or the heterogeneity of astrocytes may play a role in this phenomenon.

## NSC-sEVs and microglia

Microglia, as resident macrophages of the central nervous system, play crucial roles in various processes essential for brain development and maintenance under both physiological and pathological conditions (96). In a state of homeostasis, microglia exhibit longevity and are sustained through local proliferation independent of external sources, whereas in instances of disease, they can undergo rapid proliferation and alter their gene expression profile and morphology. The activation of microglia is influenced by various stimulators or molecules, such as NSC-sEVs. For instance, Morton et al. found that the presence of NSC-sEVs within the subventricular zone prompted neighboring microglia to undergo a phenotypic shift towards a CD11b-positive round morphology upon internalization. Furthermore, the uptake of NSC-sEVs by microglia led to a pronounced activation of their transcriptional network, establishing a feedback loop that ultimately suppressed the proliferation of neural stem cells. Consequently, NSC-sEVs, acting as morphogen-associated molecular patterns, exert a crucial influence on the regulation of microglial morphology and function (97). Moreover, the authors’ research revealed that NSC-sEVs exhibit high expression levels of miR-9, Let-7, miR-26, and miR-181. Prior investigations have shown that these specific microRNAs play a role in regulating microglial morphology and physiological functions (98–101). Recent research has shown that NSC-sEVs have a significant inhibitory effect on inflammatory factors and can alleviate inflammation by suppressing microglial activation in neurodegenerative diseases (102). For instance, Smith et al. observed a notable reduction in the number of activated microglia induced by radiation following treatment with NSC-sEVs, leading to a decrease in neuroinflammation and a pronounced neuroprotective outcome (103). Similarly, Peng et al. demonstrated that treatment with NSC-sEVs resulted in a decrease in the expression of pro-inflammatory cytokines TNF-α, IL-1β, and IL-6, mitigated the inflammatory response induced by OGD/R (oxygen glucose deprivation/reperfusion) in microglia, and improved the survival of microglia in the OGD/R model (104). These findings indicate that NSC-sEVs are significant in the modulation of neuroinflammation driven by microglia.

## NSC-sEVs in neurological diseases

Based on their physicochemical properties and biological functions, NSC-sEVs have important applications in neurological diseases (**Table 1**) (25, 67). The use of NSC-sEVs for the treatment of neurological disorders has several advantages over transplanted NSC treatments: (1) sEVs can target different areas of the brain through intranasal administration (114); (2) sEVs are not nucleated cells that do not replicate, and there is little risk of tumor or malignant transformation after treatment with sEVs (28); (3) sEVs have better biocompatibility, low immunogenicity, and cross the blood-brain barrier as natural vesicles (115); (4) sEVs can be preserved for a certain period while maintaining relative stability in their structure and function under the conditions of rapid freezing at -80°C and prevention of repeated freeze-thaw cycles. Notably, the incorporation of cryoprotectants such as dimethyl sulfoxide (DMSO), glycerol, and trehalose markedly enhances the stability of sEVs (116). Additionally, their exceptional stability during transport significantly enhances their convenience and practicality for therapeutic applications (117). Therefore, NSC-sEVs provide a non-cellular therapy for the treatment of neurological disorders without significant side effects.

**Table 1:** NSC-sEVs in neurological diseases.

Disease	Animal/cell model	Cargos	Targets	Function	References
Stroke	MCAO model	let-7g-5p/miR-99a-5p/let-7i-5p/miR-139-5p/miR-21-5p/let-7b-5p		Enhanced systemic immune responses; inhibit microglia expression and reduce inflammation; promote endogenous NSC differentiation and reduce NSC apoptosis.	(66, 105–108)
AD	5×FAD mice model/B6;C3-Tg mice model/SH-SY5Y/PSAPP-Tg	miR-138-5p	SIRT1/BACE1/PESN1/Tau	Reduces Aβ plaques; decreases microglia activation and attenuates inflammatory response; enhances mitochondrial function.	(56, 109–111)
PD	SH‐SY5Y cells, 6‐OHDA induced PD mice model	hsa-mir-17/hsa-mir-183/hsa-mir-20a/hsa-182/hsa-mir-155		Attenuates the production of reactive oxygen species, reduces the levels of pro-inflammatory cytokines and chemokines, decreases oxidative stress, and alleviates neuronal death	(57)
SCI	SCI rat model/PC12 cells/SCMEC	14-3-3p/miR-219a-2-3p	Beclin-1/YY1/VEGF-A/FTY720	Inhibits neuroinflammation, promotes autophagy, and inhibits neuronal apoptosis; promotes angiogenic activity of SCMECs.	(112, 113) (73, 75)

### Stroke

Stroke is a prominent contributor to mortality and disability on a global scale (118). Presently, tissue-type fibrinogen activator stands as the sole pharmacological intervention approved for stroke management. Nevertheless, its applicability is limited to a specific cohort of patients who receive timely hospitalization (119). The majority of stroke survivors necessitate prolonged rehabilitation and ongoing pharmacotherapy. Consequently, there exists a pressing demand for an alternative therapeutic modality capable of ameliorating cerebral injury in individuals afflicted by stroke. NSC grafts exhibit distinctive neuroprotective properties in central nervous system disorders, and they are capable of produce anti-inflammatory and neurotrophic effects through the secretion of sEVs (120). The administration of NSC-sEVs represents a highly promising therapeutic approach, particularly in the context of neurorepair following stroke, an area that has been extensively researched. For example, in their study, Webb et al. observed that in a mouse thromboembolic stroke model, NSC-sEVs facilitated an augmentation of M2-type macrophages and regulatory T cells while diminishing pro-inflammatory Th17 cells, thereby mitigating the progression of detrimental responses following stroke. Furthermore, NSC-sEVs bolstered the reparative systemic immune response and mitigated neurological damage, exhibiting greater therapeutic effectiveness in comparison to mesenchymal stem cell-derived sEVs (66). Subsequently, the therapeutic efficacy of NSC-sEVs was further evidenced in a porcine model of MCAO, resulting in reductions in brain lesion volume, brain swelling, and cerebral edema, along with enhancements in locomotor activity and expedited recovery of spatiotemporal gait parameters (105). Despite the lack of precise understanding of the underlying molecular pathways, NSC-sEVs exhibit beneficial and neuroprotective properties in experimental stroke scenarios (105). In a separate investigation, scientists administered NSC-sEVs intravenously to MCAO mouse models and observed a decrease in the expression of pro-inflammatory cytokines such as TNF-α, IL-1β, and IL-6 within ischemic regions, as well as a reduction in microglial activation, thereby suppressing inflammatory responses (106). Analysis of biological data revealed that NSC-sEVs exhibited high expression levels of let-7g-5p, miR-99a-5p, let-7i-5p, miR-139-5p, miR-21-5p, and let-7b-5p. It has been previously demonstrated that NPC-sEVs play a significant biological role through their contents, which may include protective RNA such as let-7 that can exert therapeutic effects by reducing microglia activation and inhibiting inflammatory responses (97). Furthermore, NSC-sEVs have been shown to have a dose-dependent therapeutic effect in a mouse MCAO model, leading to reductions in dyskinesia, promotion of nerve regeneration, and enhancement of axonal plasticity (107). Additionally, NPC-sEVs have been found to reverse post-ischemic peripheral immunosuppression and elevate the expression levels of B and T lymphocytes in the bloodstream (107). The administration of NSC-sEVs post-stroke demonstrates notable neuroprotective effects, motor function preservation, and recovery (66, 105–107). These non-cellular therapies exhibit promising potential for stroke treatment due to their high biocompatibility and ability to traverse biological barriers (115, 117, 121). Recent research by Zhu et al. involved encapsulating brain-derived neurotrophic factor (BDNF) within human NSC-sEVs to create engineered sEVs, highlighting the emerging role of sEVs as drug delivery systems (108). In both the rat MCAO model and the *in vitro* hydrogen peroxide-induced NSCs oxidative stress model, engineered sEVs demonstrated the ability to suppress microglial expression and decrease inflammation, thereby promoting a conducive microenvironment for the differentiation of endogenous NSCs into neurons (108). These studies are well documented that the administration of NSC-sEVs post-stroke yielded notable neuroprotective effects and modest enhancements in stroke outcomes.

### Alzheimer’s disease

Alzheimer’s disease (AD) is a progressive neurodegenerative disease characterized clinically by memory loss and cognitive decline and pathologically by neuronal fiber tangles formed by pathological accumulation of Aβ and phosphorylation of tau protein (122, 123). AD is also associated with neuronal death, loss of synapses, and brain damage (124, 125). Currently, there is a lack of effective treatment options for the prevention and cure of AD. Recent research has highlighted the potential therapeutic role of NSC-sEVs in AD. For instance, Apodaca et al. demonstrated that NSC-sEVs can mitigate Aβ accumulation by modulating microglia activation and suppressing inflammatory responses, thus ameliorating the pathological progression of AD. Additionally, NSC-sEVs were found to enhance synaptophysin levels in the brains of AD mice. This mechanism of action contributed to the restoration of memory consolidation and effectively reduced anxiety-related behavioral manifestations (109). The therapeutic efficacy of NSC-sEVs in AD was also confirmed in a study conducted by Li et al. The administration of NSC-sEVs via bilateral ventricular injection in APP/PS1(B6; C3-Tg) mice attenuated AD-related spatial learning and memory deficits, as well as increased expression levels of mitochondrial function-related factors (PGC1α, NRF1, NRF2, and Fis1) and synapse-associated proteins (synaptophysin, PSD95, MAP2). This led to neuroprotective and reparative effects. Furthermore, NSC-sEVs activated SIRT1, which in turn reduced inflammatory responses and improved AD pathology (110, 126). In a separate investigation, it was determined that NSC-sEVs have the ability to promote amyloid precursor protein (APP) processing via the non-amyloidogenic pathway. This process effectively hinders the enzymatic activity of β-secretase (BACE1) and γ-secretase (PSEN1), consequently leading to a notable reduction in Aβ production in AD (111). Interestingly, the protective effects of NSC-sEVs against AD can be augmented by the use of Chinese traditional medicine. For example, Catalpol, a water-soluble bioactive compound extracted from Rehmannia root, exhibits neuroprotective properties including antioxidative and anti-apoptotic effects, thereby playing a critical protective role in AD (127). Recent research has demonstrated that NSC-sEVs treated with Catalpol exhibit increased expression of miR-138-5p, which targets Tau and inhibits its accumulation, thereby impeding the progression of AD (56). These results underscore the significant potential of NSC-sEVs in the therapeutic management of AD.

### Parkinson’s disease

Parkinson’s disease (PD) is a progressive neurological disorder marked by the degenerative loss of nigrostriatal dopaminergic neurons and the accumulation of alpha-synuclein leading to the formation of Lewy bodies (128, 129). The etiology of PD remains unclear, however, mitochondrial dysfunction, oxidative stress, impaired axonal transport, neuroinflammation, and dysregulation of α-synuclein are implicated in the pathogenesis of the disease (128, 130, 131). Similar to AD, there is currently no definitive cure for PD, with stem cell therapy and gene therapy representing the primary treatment modalities (129). sEVs, a crucial element of the stem cell secretome, exhibit significant potential for the treatment of PD. For example, Lee et al. conducted a study on the impact of human NSC-sEVs in a PD model (57). In the *in vitro* PD model constructed using 6-OHDA-treated SH-SY5Y cells, these sEVs demonstrated significant neuroprotective effects by reducing the generation of reactive oxygen species (ROS) and lowering the levels of pro-inflammatory cytokines and chemokines, thereby effectively mitigating neuronal loss. Furthermore, small RNA sequencing of these sEVs revealed high expression levels of five specific miRNAs (hsa-mir-17, hsa-mir-183, hsa-mir-20a, hsa-182, hsa-mir-155). Previous studies suggested that these miRNAs are involved in various biological processes such as neurogenesis, cell differentiation, and immune response (57). For instance, hsa-miR-183-5p and hsa-miR-182-5p have been shown to mimic the effects of glial cell-derived neurotrophic factor, leading to a reduction in apoptosis of dopaminergic neurons in PD (132). Moreover, NSC-sEVs have been found to protect degenerating neurons by exerting anti-apoptotic, anti-oxidative stress, and anti-inflammatory effects through the miRNA cargo they contain. For instance, it has been previously mentioned that miR-26a-5p is a highly expressed miRNA in NSC-sEVs. Research conducted by Chen M et al. has demonstrated that miR-26a-5p within sEVs can promote the proliferation of LPS-treated rat pheochromocytoma cell line PC12-derived cells and inhibit their apoptosis, thereby contributing to the survival of PC12 cells (133). This suggests that NSC-sEVs could offer promising therapeutic options for PD (57). Nevertheless, further research is necessary to elucidate the underlying mechanisms involved in the treatment with NSC-sEVs.

### Spinal Cord Injury

Spinal Cord Injury (SCI) is a neurological disorder with a high disability rate, for which there is no effective treatment, and which places a heavy burden on families and society (134, 135). SCI produces a range of pathological responses, including apoptosis, inflammation, oxidative stress, vascular rupture, and glial scar formation, which pose a significant clinical treatment challenge (136, 137). Multiple studies have examined the potential therapeutic effects of NSC-sEVs in the context of SCI. The observed effects of NSC-sEVs include anti-inflammatory and anti-oxidative properties, inhibition of neuroinflammation, suppression of neuronal apoptosis, and promotion of endogenous NSCs differentiation through the delivery of miRNAs and proteins. For instance, Rong et al. demonstrated that NSC-sEVs can mitigate neuronal apoptosis, suppress neuroinflammation, and enhance functional recovery by inducing autophagy in the rat SCI model (136). Furthermore, Rong and colleagues elucidated that the underlying mechanism of NSC-sEVs action involves upregulating autophagy through targeted delivery of Beclin-1 to 14-3-3t, thereby attenuating SCI-induced apoptosis and neuroinflammation (112). In a separate investigation, NSC-sEVs treated with insulin-like growth factors were observed to suppress neuroinflammation and apoptosis while enhancing neuroprotection by upregulating miR-219a-2-3p, which in turn downregulated YY1 expression and mitigated inflammatory NF-κB pathway activation (113). The neuroprotective effects of NSC-sEVs on SCI are believed to be due to their facilitation of angiogenesis in addition to their capacity to reduce apoptosis and inhibit neuroinflammation. For instance, Dong et al. demonstrated that NSC-sEVs exhibit elevated levels of vascular endothelial growth factor-A (VEGF-A), thereby enhancing the angiogenic capabilities of spinal cord microvascular endothelial cells (SCMECs) and facilitating the regeneration of microvasculature and functional recovery following SCI (75). FTY720, a functional antagonist of the sphingosine-1-phosphate receptor, has been shown to promote microvascular remodeling (138). Chen et al. discovered that FTY720-loaded NSC-sEVs can stimulate microvascular remodeling and suppress neuronal cell apoptosis by modulating the PTEN/AKT signaling pathway (73). These findings offer novel therapeutic approaches for the management of SCI. In conclusion, the results of this study indicate that NSC-sEVs hold potential as a non-cellular therapeutic approach for neurological disorders. It is important to note, however, that while NSC-sEVs have demonstrated efficacy in multiple disease models, further research is needed to fully elucidate their therapeutic mechanisms.

## Optimization strategies for NSC-sEVs treatment

To date, the specific mechanism of action of NSC-sEVs has not been fully elucidated and their biological effects in neurological physiopathological models remain controversial. For example, some experiments have shown that NSC-sEVs inhibit microglial activation, but other studies have reported that they can induce the transition of microglia to a CD11b/Iba1-activated morphology (97). In addition, techniques to identify, isolate and purify sEVs from heterogeneous neural stem cell sources are lacking. Significant differences in miRNAs, protein profiles and functional activities in sEVs secreted by NSCs from different donors (age, gender, genetic background) and differentiation states (quiescent, proliferative or differentiated) lead to unpredictable therapeutic outcomes (82). In particular, NSCs cultured *in vitro* for long periods can develop genetic drift, leading to an increased risk of clinical application of their secreted sEVs (139). Meanwhile, some studies have reported that NSC-sEVs may carry abnormal signaling molecules that may pose potential safety hazards during treatment. For example, studies have reported that the anionic phospholipids of NSC-sEVs may interact with some viruses, allowing receptor-independent entry of the virus into the cell (140). Other studies have similarly uncovered that during the application of NSC-sEVs in neurogenesis/neurorepair processes, these vesicles may induce aberrant proliferation of non-target cells through the conveyance of pro-proliferative signals—such as factors linked to the Wnt/β-catenin pathway (141). In the future, it is necessary to break the bottleneck of contradiction and reproducibility in current research by standardizing the source of NSC-sEVs and refining the analysis of the molecular mechanism, so as to lay the foundation for the treatment of NSC-sEVs in the nervous system.

**Figure 3:**
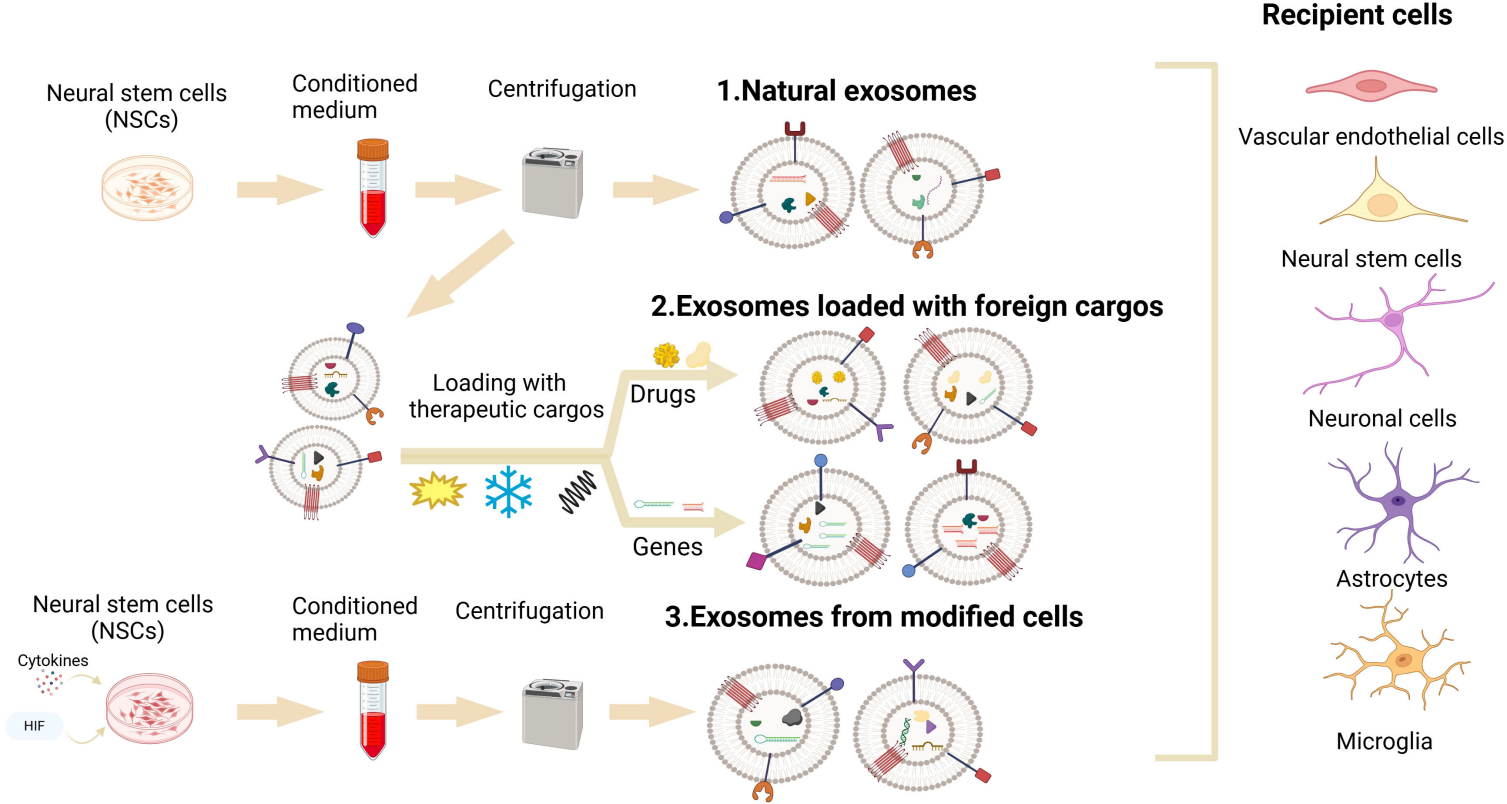
NSC-sEVs’ engineering: Two key engineering techniques for optimization of the therapeutic potential of NSC-sEVs for neurological diseases. One involves the administration of exogenous factors (hypoxia, inflammation, cytokines) to directly modify NSCs parental cells to induce them to secrete NSC-sEVs with specific components. Another approach requires the isolation of NSC-sEVs, followed by the loading of various cargoes (proteins, RNA, DNA, and drugs) into NSC-sEVs using co-incubation, electroporation, sonication, freeze-thaw cycling, extrusion, saponification, and chemical transfection techniques.

Despite these challenges, NSC-sEVs still hold great potential in the treatment of neurological diseases, and current research is exploring ways to overcome these limitations. NSC-sEVs are currently under development as drug delivery systems for the treatment of neurological diseases, yet they are hindered by inherent limitations such as low bioactivity, weak targeting ability, and short half-life *in vivo* (142, 143). To enhance the therapeutic potential of NSC-sEVs, the utilization of engineering techniques for their modification has emerged as a promising strategy. Currently, there exist two primary approaches for the engineering of NSC-sEVs: one involves direct modification of NSCs parental cells to induce the secretion of NSC-sEVs with specific compositions, while the other method entails the isolation of NSC-sEVs followed by subsequent modifications (**Figure 3**). These methodologies address the shortcomings related to yield, bioactivity, targeting capabilities, and circulating half-life of natural NSC-sEVs. On the one hand, the biological activity of NSC-sEVs derived from parental NSCs can be modulated through the administration of exogenous factors such as hypoxia, inflammation, cytokines (55, 113, 144). For instance, Zhang et al. demonstrated that NSC-sEVs produced following IFN-γ stimulation exhibited increased efficacy in mitigating apoptosis and inflammation in ischemic stroke models compared to unstimulated NSCs (55). Chen et al. have also demonstrated the role of NSC-sEVs, produced after IFN-γ stimulation, in the attenuation of neuroinflammation as well as neuronal damage (145). Ma et al. further illustrated that the therapeutic potential of NSC-sEVs could be augmented through treatment with insulin-like growth factor (113). On the other hand, in addition to modulating parental NSCs to enhance biological activity, the loading of exogenous cargoes directly into NSC-sEVs represents a promising strategy for further enhancing their therapeutic effects. Various cargo loading methods, including co-incubation, electroporation, ultrasonication, freeze-thaw cycles, extrusion, saponification, and chemical transfection, have been utilized in research (146). Notably, direct loading of cargo has shown promising results in enhancing bioactivity. For instance, Zhu et al. demonstrated enhanced anti-apoptotic and pro-differentiation effects by co-incubating BDNF with hNSC-sEVs in an ischemic stroke model (108). Similarly, Qian et al. successfully loaded miR-124-3p into NSC-sEVs via electroporation, leading to inhibition of glioma growth by targeting Flotillin2 in a glioma model (147). These findings indicate that augmenting the bioactivity of NSC-sEVs may amplify their therapeutic efficacy.

Despite the ability of sEVs to traverse the blood-brain barrier, studies have demonstrated that following injection of sEVs derived from various cell types into mice, these vesicles primarily accumulate in the liver, spleen, gastrointestinal tract, and lungs, with only a minimal fraction reaching the brain (148). In order to optimize the therapeutic potential of NSC-sEVs for neurological disorders, strategies for enhancing the brain-specific targeting of these vesicles have been developed through various engineering techniques. The utilization of Lamp2b-RVG in the modification of brain targeting of sEVs is prevalent in current research. Lamp2b, a lysosome-associated membrane glycoprotein, is a prominent membrane-localized protein found on sEVs, while RVG, a rabies virus glycoprotein, is a specific neuronal peptide that binds to acetylcholine receptors (143, 149, 150). Various studies have demonstrated that Lamp2b-RVG targeted sEVs have the ability to reach various cell types within the brain, including neurons, astrocytes, microglia, and oligodendrocytes, and can elicit therapeutic effects through the delivery of encapsulated cargo (151, 152). In addition to modifying parental cells to enhance targeting ability, sEVs can also be directly targeted. Tian et al. developed a recombinant fusion protein incorporating an arginine-glycine-aspartate (RGD)-4C peptide (ACDCRGDCFC) fused with the phosphatidylserine binding domain of lactadherin (C1C2), which effectively binds to the membrane of sEVs. This engineered protein attaches to the surface of sEVs and targets αvβ3 to suppress post-stroke inflammation, exhibiting therapeutic benefits in stroke treatment (106). Through engineering techniques, the brain targeting of sEVs can be enhanced to improve their therapeutic efficacy. In summary, the engineering of NSC-sEVs has the potential to address the limitations of natural sEVs, leading to improved therapeutic efficacy. Furthermore, the utilization of NSC-sEVs in drug delivery systems for the treatment of neurological disorders represents a promising avenue for non-cellular therapy.

Although the aforementioned engineering modifications have shown great promise in advancing the clinical application process of NSC-sEVs, it is important to recognize that significant technical bottlenecks still remain in key areas such as establishing standards for large-scale production, pharmacodynamics, and safety assessment. Currently, the technologies for separating sEVs have diversified (including ultracentrifugation based on physical parameters, biomolecule-dependent chemical affinity, and microfluidic microarrays), yet there is still a lack of unified quality control standards and standardized procedures for large-scale separation, production, and storage of sEVs (153). In addition, there is still a large gap in the area of efficacy assessment and safety studies of sEVs-based therapeutic regimens: On the one hand, a standardized protocol for quantifying the dose-response relationship (including key parameters such as effective dose, route and frequency of administration, and stability of clinical application) has not yet been established (154); On the other hand, the potential off-target effects and immunogenicity risks that may be caused by the biologically active molecules, such as nucleic acids and proteins, carried in the vesicle need to be systematically evaluated (155). Future research should aim to establish a standardized system for the clinical treatment of NSC-sEVs, focusing on the development of a standardized preparation process as well as precise and quantitative therapeutic methods, in order to give NSC-sEVs a broader development perspective.

## Discussion

The advancement of regenerative medicine has led to the recognition of NSC-sEVs as promising candidates for the next wave of non-cellular therapies in the field of neurological diseases. Currently, the clinical translation of NSC-sEVs remains in the exploratory phase, with evidence limited to basic research and preclinical studies, and has not yet advanced to large-scale clinical trials. However, in the future, with deeper research and technological advances, NSC-sEVs are expected to play an important role in the treatment of neurological diseases. NSC-sEVs demonstrate significant potential as drug delivery vehicles for the treatment of such conditions, owing to their biocompatibility, low immunogenicity, and ability to traverse the blood-brain barrier. Our review suggests that, in normal physiological circumstances, NSC-sEVs play a crucial regulatory role in the growth and functional upkeep of the central nervous system. In the context of neurological disorders, NSC-sEVs have demonstrated the capacity to mitigate neuronal apoptosis, dampen neuroinflammation, mitigate oxidative stress, enhance synaptic function, and stimulate endogenous NSC differentiation via their cargo contents, thereby impeding disease advancement. Additionally, we examine various engineering methodologies aimed at harnessing NSC-sEVs as vehicles for drug delivery in the management of neurological disorders. These approaches serve to enhance the therapeutic efficacy and targeting precision of NSC-sEVs, thereby facilitating their clinical translation for disease intervention. In summary, NSC-sEVs represent a promising non-cellular therapeutic modality for the treatment of neurological disorders, offering potential advantages over NSCs transplantation by mitigating associated challenges.

Despite the considerable promise of NSC-sEVs in the management of neurological disorders, notable obstacles persist. These include an incomplete understanding of the therapeutic mechanisms of NSC-sEVs in neurological disorders, necessitating further investigation. Additionally, the absence of reliable and efficient techniques for isolating and purifying sEVs hinders their clinical application in treating neurological diseases. Furthermore, the optimization of target modification strategies is imperative to mitigate off-target effects and augment the therapeutic efficacy of NSC-sEVs. Finally, standardizing the source of NSC-sEVs is essential for assessing the immunogenicity and toxicity of these vesicles from various origins, which may exhibit specific side effects. It is indisputable that NSC-sEVs exhibit significant promise as non-cellular therapeutic agents for addressing neurological disorders. Enhanced understanding of the molecular pathways underlying the efficacy of NSC-sEVs in neurological disease treatment is crucial for facilitating the translation of these findings into clinical practice.

## References

[1] Collaborators, G. N. Global, regional, and national burden of neurological disorders 1990-2016: a systematic analysis for the Global Burden of Disease Study 2016. Lancet Neurol. (2019) 18(5):459–80. doi: 10.1016/S1474-4422(18)30499-X PMC6459001 30879893

[2] GBD 2017 Disease and Injury Incidence and Prevalence Collaborators. Global, regional, and national incidence, prevalence, and years lived with disability for 354 diseases and injuries for 195 countries and territories 1990-2017: a systematic analysis for the Global Burden of Disease Study 2017. Lancet. (2018) 392(10159):1789–858. doi: 10.1016/S0140-6736(18)32279-7 PMC6227754 30496104

[3] Wang L Zhang L. Circulating exosomal miRNA as diagnostic biomarkers of neurodegenerative diseases. Front Mol Neurosci. (2020) 13:53. doi: 10.3389/fnmol.2020.00053 32351363 PMC7174585

[4] Fayazi N Sheykhhasan M Soleimani Asl S Najafi R. Stem cell-derived exosomes: a new strategy of neurodegenerative disease treatment. Mol Neurobiol. (2021) 58:3494–514. doi: 10.1007/s12035-021-02324-x PMC7981389 33745116

[5] Courtine G Sofroniew MV. Spinal cord repair: advances in biology and technology. Nat Med. (2019) 25:898–908. doi: 10.1038/s41591-019-0475-6 31160817

[6] Thier M Wörsdörfer P Lakes YB Gorris R Herms S Opitz T. Direct conversion of fibroblasts into stably expandable neural stem cells. Cell Stem Cell. (2012) 10:473–9. doi: 10.1016/j.stem.2012.03.003 22445518

[7] Tang Y Yu P Cheng L. Current progress in the derivation and therapeutic application of neural stem cells. Cell Death Dis. (2017) 8:e3108. doi: 10.1038/cddis.2017.504 29022921 PMC5682670

[8] Kumamaru H Kadoya K Adler AF Takashima Y Graham L Coppola G. Generation and post-injury integration of human spinal cord neural stem cells. Nat Methods. (2018) 15:723–31. doi: 10.1038/s41592-018-0074-3 30082899

[9] Beattie R Hippenmeyer S. Mechanisms of radial glia progenitor cell lineage progression. FEBS Lett. (2017) 591:3993–4008. doi: 10.1002/1873-3468.12906 29121403 PMC5765500

[10] Madrazo I Kopyov O Ávila-Rodríguez MA Ostrosky F Carrasco H Kopyov A. Transplantation of Human Neural Progenitor Cells (NPC) into Putamina of Parkinsonian Patients: A Case Series Study, Safety and Efficacy Four Years after Surgery. Cell Transplant. (2019) 28:269–85. doi: 10.1177/0963689718820271 PMC6425108 30574805

[11] Ryu S Lee SH Kim SU Yoon BW. Human neural stem cells promote proliferation of endogenous neural stem cells and enhance angiogenesis in ischemic rat brain. Neural Regener Res. (2016) 11:298–304. doi: 10.4103/1673-5374.177739 PMC4810995 27073384

[12] Ager RR Davis JL Agazaryan A Benavente F Poon WW Laferla FM. Human neural stem cells improve cognition and promote synaptic growth in two complementary transgenic models of Alzheimer’s disease and neuronal loss. Hippocampus. (2015) 25:813–26. doi: 10.1002/hipo.22405 PMC4722865 25530343

[13] Andres RH Horie N Slikker W Keren-Gill H Zhan K Sun G. Human neural stem cells enhance structural plasticity and axonal transport in the ischaemic brain. Brain. (2011) 134:1777–89. doi: 10.1093/brain/awr094 PMC3102243 21616972

[14] Boese AC Le QE Pham D Hamblin MH Lee JP. Neural stem cell therapy for subacute and chronic ischemic stroke. Stem Cell Res Ther. (2018) 9:154. doi: 10.1186/s13287-018-0913-2 29895321 PMC5998588

[15] Gong Z Lei D Wang C Yu C Xia K Shu J. Bioactive elastic scaffolds loaded with neural stem cells promote rapid spinal cord regeneration. ACS Biomater Sci Eng. (2020) 6:6331–43. doi: 10.1021/acsbiomaterials.0c01057 33449647

[16] Pöyhönen S Er S Domanskyi A Airavaara M. Effects of neurotrophic factors in glial cells in the central nervous system: expression and properties in neurodegeneration and injury. Front Physiol. (2019) 10:486. doi: 10.3389/fphys.2019.00486 31105589 PMC6499070

[17] Shoemaker LD Kornblum HI. Neural stem cells (NSCs) and proteomics. Mol Cell Proteomics. (2016) 15:344–54. doi: 10.1074/mcp.o115.052704 PMC4739658 26494823

[18] Ni W Ramalingam M Li Y Park JH Dashnyam K Lee JH. Immunomodulatory and anti-inflammatory effect of neural stem/progenitor cells in the central nervous system. Stem Cell Rev Rep. (2023) 19(4):866–85. doi: 10.1007/s12015-022-10501-1 36650367

[19] Willis CM Nicaise AM Peruzzotti-Jametti L Pluchino S. The neural stem cell secretome and its role in brain repair. Brain Res. (2020) 1729:146615. doi: 10.1016/j.brainres.2019.146615 31863730

[20] Zhong L Wang J Wang P Liu X Liu P Cheng X. Neural stem cell-derived exosomes and regeneration: cell-free therapeutic strategies for traumatic brain injury. Stem Cell Res Ther. (2023) 14:198. doi: 10.1186/s13287-023-03409-1 37553595 PMC10408078

[21] Patil M Henderson J Luong H Annamalai D Sreejit G Krishnamurthy P. The art of intercellular wireless communications: exosomes in heart disease and therapy. Front Cell Dev Biol. (2019) 7:315. doi: 10.3389/fcell.2019.00315 31850349 PMC6902075

[22] Wiklander OPB Brennan M Lötvall J Breakefield XO El Andaloussi S. Advances in therapeutic applications of extracellular vesicles. Sci Transl Med. (2019) 11(492):eaav8521. doi: 10.1126/scitranslmed.aav8521 31092696 PMC7104415

[23] Harding C Heuser J Stahl P. Receptor-mediated endocytosis of transferrin and recycling of the transferrin receptor in rat reticulocytes. J Cell Biol. (1983) 97:329–39. doi: 10.1083/jcb.97.2.329 PMC2112509 6309857

[24] Kalluri R Lebleu VS. The biology, function, and biomedical applications of exosomes. Science. (2020) 367(6478):eaau6977. doi: 10.1126/science.aau6977 32029601 PMC7717626

[25] Mathieu M Martin-Jaular L Lavieu G Théry C. Specificities of secretion and uptake of exosomes and other extracellular vesicles for cell-to-cell communication. Nat Cell Biol. (2019) 21:9–17. doi: 10.1038/s41556-018-0250-9 30602770

[26] Théry C Witwer KW Aikawa E Alcaraz MJ Anderson JD Andriantsitohaina R. Minimal information for studies of extracellular vesicles 2018 (MISEV2018): a position statement of the International Society for Extracellular Vesicles and update of the MISEV2014 guidelines. J Extracell Vesicles. (2018) 7(1):1535750. doi: 10.1080/20013078.2018.1535750 30637094 PMC6322352

[27] Xia X Wang Y Zheng JC. Extracellular vesicles, from the pathogenesis to the therapy of neurodegenerative diseases. Transl Neurodegener. (2022) 11:53. doi: 10.1186/s40035-022-00330-0 36510311 PMC9743667

[28] Van Niel G D’angelo G Raposo G. Shedding light on the cell biology of extracellular vesicles. Nat Rev Mol Cell Biol. (2018) 19:213–28. doi: 10.1038/nrm.2017.125 29339798

[29] Sharma P Mesci P Carromeu C Mcclatchy DR Schiapparelli L Yates JR 3rd. Exosomes regulate neurogenesis and circuit assembly. Proc Natl Acad Sci U.S.A. (2019) 116:16086–94. doi: 10.1073/pnas.1902513116 PMC6689941 31320591

[30] Joshi BS Zuhorn IS. Heparan sulfate proteoglycan-mediated dynamin-dependent transport of neural stem cell exosomes in an *in vitro* blood-brain barrier model. Eur J Neurosci. (2021) 53:706–19. doi: 10.1111/ejn.14974 PMC7891616 32939863

[31] Holm MM Kaiser J Schwab ME. Extracellular vesicles: multimodal envoys in neural maintenance and repair. Trends Neurosci. (2018) 41:360–72. doi: 10.1016/j.tins.2018.03.006 29605090

[32] Garrus JE Von Schwedler UK Pornillos OW Morham SG Zavitz KH Wang HE. Tsg101 and the vacuolar protein sorting pathway are essential for HIV-1 budding. Cell. (2001) 107:55–65. doi: 10.1016/s0092-8674(01)00506-2 11595185

[33] Agromayor M Soler N Caballe A Kueck T Freund SM Allen MD. The UBAP1 subunit of ESCRT-I interacts with ubiquitin via a SOUBA domain. Structure. (2012) 20:414–28. doi: 10.1016/j.str.2011.12.013 PMC3314968 22405001

[34] Teo H Perisic O González B Williams RL. ESCRT-II, an endosome-associated complex required for protein sorting: crystal structure and interactions with ESCRT-III and membranes. Dev Cell. (2004) 7(4):559–69. doi: 10.1016/j.devcel.2004.09.003 15469844

[35] Larios J Mercier V Roux A Gruenberg J. ALIX- and ESCRT-III-dependent sorting of tetraspanins to exosomes. J Cell Biol. (2020) 219(3):e201904113. doi: 10.1083/jcb.201904113 32049272 PMC7054990

[36] Raiborg C Wesche J Malerød L Stenmark H. Flat clathrin coats on endosomes mediate degradative protein sorting by scaffolding Hrs in dynamic microdomains. J Cell Sci. (2006) 119:2414–24. doi: 10.1242/jcs.02978 16720641

[37] Tschuschke M Kocherova I Bryja A Mozdziak P Angelova Volponi A Janowicz K. Inclusion biogenesis, methods of isolation and clinical application of human cellular exosomes. J Clin Med. (2020) 9(2):436. doi: 10.3390/jcm9020436 32041096 PMC7074492

[38] Hurley JH Hanson PI. Membrane budding and scission by the ESCRT machinery: it’s all in the neck. Nat Rev Mol Cell Biol. (2010) 11:556–66. doi: 10.1038/nrm2937 PMC2922035 20588296

[39] Kenific CM Zhang H Lyden D. An exosome pathway without an ESCRT. Cell Res. (2021) 31:105–6. doi: 10.1038/s41422-020-00418-0 PMC8027877 32973340

[40] Wei D Zhan W Gao Y Huang L Gong R Wang W. RAB31 marks and controls an ESCRT-independent exosome pathway. Cell Res. (2021) 31:157–77. doi: 10.1038/s41422-020-00409-1 PMC8027411 32958903

[41] Deng H Sun C Sun Y Li H Yang L Wu D. Lipid, protein, and microRNA composition within mesenchymal stem cell-derived exosomes. Cell Reprogram. (2018) 20:178–86. doi: 10.1089/cell.2017.0047 29782191

[42] Laulagnier K Javalet C Hemming FJ Chivet M Lachenal G Blot B. Amyloid precursor protein products concentrate in a subset of exosomes specifically endocytosed by neurons. Cell Mol Life Sci. (2018) 75:757–73. doi: 10.1007/s00018-017-2664-0 PMC11105273 28956068

[43] Munshi A Mehic J Creskey M Gobin J Gao J Rigg E. A comprehensive proteomics profiling identifies NRP1 as a novel identity marker of human bone marrow mesenchymal stromal cell-derived small extracellular vesicles. Stem Cell Res Ther. (2019) 10:401. doi: 10.1186/s13287-019-1516-2 31852509 PMC6921509

[44] Upadhya R Madhu LN Attaluri S Gitaí DLG Pinson MR Kodali M. Extracellular vesicles from human iPSC-derived neural stem cells: miRNA and protein signatures, and anti-inflammatory and neurogenic properties. J Extracell Vesicles. (2020) 9:1809064. doi: 10.1080/20013078.2020.1809064 32944193 PMC7480597

[45] Varderidou-Minasian S Lorenowicz MJ. Mesenchymal stromal/stem cell-derived extracellular vesicles in tissue repair: challenges and opportunities. Theranostics. (2020) 10:5979–97. doi: 10.7150/thno.40122 PMC7254996 32483432

[46] Upadhya R Madhu LN Rao S Shetty AK. Proficiency of Extracellular Vesicles From hiPSC-Derived Neural Stem Cells in Modulating Proinflammatory Human Microglia: Role of Pentraxin-3 and miRNA-21-5p. Front Mol Neurosci. (2022) 15:845542. doi: 10.3389/fnmol.2022.845542 35656007 PMC9152457

[47] Johnson TK Zhao L Zhu D Wang Y Xiao Y Oguljahan B. Exosomes derived from induced vascular progenitor cells promote angiogenesis *in vitro* and in an *in vivo* rat hindlimb ischemia model. Am J Physiol Heart Circ Physiol. (2019) 317:H765–h776. doi: 10.1152/ajpheart.00247.2019 31418583 PMC6843021

[48] Wills CA Liu X Chen L Zhao Y Dower CM Sundstrom J. Chemotherapy-induced upregulation of small extracellular vesicle-associated PTX3 accelerates breast cancer metastasis. Cancer Res. (2021) 81:452–63. doi: 10.1158/0008-5472.can-20-1976 PMC7855036 33115808

[49] Kaminska A Radoszkiewicz K Rybkowska P Wedzinska A Sarnowska A. Interaction of neural stem cells (NSCs) and mesenchymal stem cells (MSCs) as a promising approach in brain study and nerve regeneration. Cells. (2022) 11(9):1464. doi: 10.3390/cells11091464 35563770 PMC9105617

[50] Ulrich H Do Nascimento IC Bocsi J Tárnok A. Immunomodulation in stem cell differentiation into neurons and brain repair. Stem Cell Rev Rep. (2015) 11:474–86. doi: 10.1007/s12015-014-9556-6 25267435

[51] Zhang S Teo KYW Chuah SJ Lai RC Lim SK Toh WS. MSC exosomes alleviate temporomandibular joint osteoarthritis by attenuating inflammation and restoring matrix homeostasis. Biomaterials. (2019) 200:35–47. doi: 10.1016/j.biomaterials.2019.02.006 30771585

[52] Wei H Xu Y Chen Q Chen H Zhu X Li Y. Mesenchymal stem cell-derived exosomal miR-223 regulates neuronal cell apoptosis. Cell Death Dis. (2020) 11:290. doi: 10.1038/s41419-020-2490-4 32341353 PMC7184756

[53] Peng C Li Y Lu L Zhu J Li H Hu J. Efficient one-step induction of human umbilical cord-derived mesenchymal stem cells (UC-MSCs) produces MSC-derived neurospheres (MSC-NS) with unique transcriptional profile and enhanced neurogenic and angiogenic secretomes. Stem Cells Int. (2019) 2019:9208173. doi: 10.1155/2019/9208173 31933651 PMC6942888

[54] Cossetti C Iraci N Mercer TR Leonardi T Alpi E Drago D. Extracellular vesicles from neural stem cells transfer IFN-γ via Ifngr1 to activate Stat1 signaling in target cells. Mol Cell. (2014) 56:193–204. doi: 10.1016/j.molcel.2014.11.009 25242146 PMC4578249

[55] Zhang G Zhu Z Wang H Yu Y Chen W Waqas A. Exosomes derived from human neural stem cells stimulated by interferon gamma improve therapeutic ability in ischemic stroke model. J Adv Res. (2020) 24:435–45. doi: 10.1016/j.jare.2020.05.017 PMC7289755 32551140

[56] Meng S Chen H Deng C Meng Z. Catalpol mitigates alzheimer’s disease progression by promoting the expression of neural stem cell exosomes released miR-138-5p. Neurotox Res. (2023) 41:41–56. doi: 10.1007/s12640-022-00626-z 36595161 PMC9944361

[57] Lee EJ Choi Y Lee HJ Hwang DW Lee DS. Human neural stem cell-derived extracellular vesicles protect against Parkinson’s disease pathologies. J Nanobiotechnology. (2022) 20:198. doi: 10.1186/s12951-022-01356-2 35468855 PMC9040239

[58] Liu X Zhang G Wei P Hao L Zhong L Zhong K. 3D-printed collagen/silk fibroin/secretome derived from bFGF-pretreated HUCMSCs scaffolds enhanced therapeutic ability in canines traumatic brain injury model. Front Bioeng Biotechnol. (2022) 10:995099. doi: 10.3389/fbioe.2022.995099 36091465 PMC9449499

[59] Zhang S Chuah SJ Lai RC Hui JHP Lim SK Toh WS. MSC exosomes mediate cartilage repair by enhancing proliferation, attenuating apoptosis and modulating immune reactivity. Biomaterials. (2018) 156:16–27. doi: 10.1016/j.biomaterials.2017.11.028 29182933

[60] Du W Zhang K Zhang S Wang R Nie Y Tao H. Enhanced proangiogenic potential of mesenchymal stem cell-derived exosomes stimulated by a nitric oxide releasing polymer. Biomaterials. (2017) 133:70–81. doi: 10.1016/j.biomaterials.2017.04.030 28433939

[61] Li Q Zhang F Fu X Han N. Therapeutic potential of mesenchymal stem cell-derived exosomes as nanomedicine for peripheral nerve injury. Int J Mol Sci. (2024) 25(14):7882. doi: 10.3390/ijms25147882 39063125 PMC11277195

[62] Gomes DE Witwer KW. L1CAM-associated extracellular vesicles: A systematic review of nomenclature, sources, separation, and characterization. J Extracell Biol. (2022) 1(3):e35. doi: 10.1002/jex2.35 35492832 PMC9045013

[63] Fauré J Lachenal G Court M Hirrlinger J Chatellard-Causse C Blot B. Exosomes are released by cultured cortical neurones. Mol Cell Neurosci. (2006) 31:642–8. doi: 10.1016/j.mcn.2005.12.003 16446100

[64] Dutta S Hornung S Taha HB Bitan G. Biomarkers for parkinsonian disorders in CNS-originating EVs: promise and challenges. Acta Neuropathol. (2023) 145:515–40. doi: 10.1007/s00401-023-02557-1 PMC10071251 37012443

[65] Lu Y Wang L Zhang M Chen Z. Mesenchymal stem cell-derived small extracellular vesicles: A novel approach for kidney disease treatment. Int J Nanomedicine. (2022) 17:3603–18. doi: 10.2147/IJN.S372254 PMC9386173 35990308

[66] Webb RL Kaiser EE Scoville SL Thompson TA Fatima S Pandya C. Human neural stem cell extracellular vesicles improve tissue and functional recovery in the murine thromboembolic stroke model. Transl Stroke Res. (2018) 9:530–9. doi: 10.1007/s12975-017-0599-2 PMC6132936 29285679

[67] Stevanato L Thanabalasundaram L Vysokov N Sinden JD. Investigation of content, stoichiometry and transfer of miRNA from human neural stem cell line derived exosomes. PloS One. (2016) 11:e0146353. doi: 10.1371/journal.pone.0146353 26752061 PMC4713432

[68] Sun Q Ma L Qiao J Wang X Li J Wang Y. MiR-181a-5p promotes neural stem cell proliferation and enhances the learning and memory of aged mice. Aging Cell. (2023) 22:e13794. doi: 10.1111/acel.13794 36797653 PMC10086527

[69] Wang L Shui X Zhang M Mei Y Xia Y Lan G. MiR-191-5p attenuates tau phosphorylation, Aβ Generation, and neuronal cell death by regulating death-associated protein kinase 1. ACS Chem Neurosci. (2022) 13:3554–66. doi: 10.1021/acschemneuro.2c00423 36454178

[70] Qi D Hou X Jin C Chen X Pan C Fu H. HNSC exosome-derived MIAT improves cognitive disorders in rats with vascular dementia via the miR-34b-5p/CALB1 axis. Am J Transl Res. (2021) 13(9):10075–93. PMC8507051 34650682

[71] Li W Shan B Cheng X He H Qin J Zhao H. circRNA Acbd6 promotes neural stem cell differentiation into cholinergic neurons via the miR-320-5p-Osbpl2 axis. J Biol Chem. (2022) 298:101828. doi: 10.1016/j.jbc.2022.101828 35305988 PMC9018392

[72] Krolak T Chan KY Kaplan L Huang Q Wu J Zheng Q. A high-efficiency AAV for endothelial cell transduction throughout the central nervous system. Nat Cardiovasc Res. (2022) 1:389–400. doi: 10.1038/s44161-022-00046-4 35571675 PMC9103166

[73] Chen J Zhang C Li S Li Z Lai X Xia Q. Exosomes derived from nerve stem cells loaded with FTY720 promote the recovery after spinal cord injury in rats by PTEN/AKT signal pathway. J Immunol Res. (2021) 2021:8100298. doi: 10.1155/2021/8100298 34337080 PMC8294984

[74] Zhang L Graf I Kuang Y Zheng X Haupt M Majid A. Neural progenitor cell-derived extracellular vesicles enhance blood-brain barrier integrity by NF-κB (Nuclear factor-κB)-dependent regulation of ABCB1 (ATP-binding cassette transporter B1) in stroke mice. Arterioscler Thromb Vasc Biol. (2021) 41:1127–45. doi: 10.1161/atvbaha.120.315031 PMC7901534 33327747

[75] Zhong D Cao Y Li CJ Li M Rong ZJ Jiang L. Neural stem cell-derived exosomes facilitate spinal cord functional recovery after injury by promoting angiogenesis. Exp Biol Med (Maywood). (2020) 245:54–65. doi: 10.1177/1535370219895491 31903774 PMC6987743

[76] Deng X Hu X Wang S Zhao H Wei Y Fu J. Neural stem cell-derived exosomes regulate cell proliferation, migration, and cell death of brain microvascular endothelial cells via the miR-9/Hes1 axis under hypoxia. Anim Model Exp Med. (2024) 7:24–35. doi: 10.1002/ame2.12394 PMC10961869 38369683

[77] Nawaz M Fatima F Vallabhaneni KC Penfornis P Valadi H Ekström K. Extracellular vesicles: evolving factors in stem cell biology. Stem Cells Int. (2016) 2016:1073140. doi: 10.1155/2016/1073140 26649044 PMC4663346

[78] Ma Y Wang K Pan J Fan Z Tian C Deng X. Induced neural progenitor cells abundantly secrete extracellular vesicles and promote the proliferation of neural progenitors via extracellular signal-regulated kinase pathways. Neurobiol Dis. (2019) 124:322–34. doi: 10.1016/j.nbd.2018.12.003 PMC6450400 30528256

[79] Yuan P Ding L Chen H Wang Y Li C Zhao S. Neural stem cell-derived exosomes regulate neural stem cell differentiation through miR-9-hes1 axis. Front Cell Dev Biol. (2021) 9:601600. doi: 10.3389/fcell.2021.601600 34055767 PMC8155619

[80] Tan SL Ohtsuka T González A Kageyama R. MicroRNA9 regulates neural stem cell differentiation by controlling Hes1 expression dynamics in the developing brain. Genes Cells. (2012) 17:952–61. doi: 10.1111/gtc.12009 23134481

[81] Ma Y Li C Huang Y Wang Y Xia X Zheng JC. Exosomes released from neural progenitor cells and induced neural progenitor cells regulate neurogenesis through miR-21a. Cell Commun Signal. (2019) 17:96. doi: 10.1186/s12964-019-0418-3 31419975 PMC6698014

[82] Zhang J Uchiyama J Imami K Ishihama Y Kageyama R Kobayashi T. Novel roles of small extracellular vesicles in regulating the quiescence and proliferation of neural stem cells. Front Cell Dev Biol. (2021) 9:762293. doi: 10.3389/fcell.2021.762293 34805169 PMC8601375

[83] Zhang Y Kim MS Jia B Yan J Zuniga-Hertz JP Han C. Hypothalamic stem cells control ageing speed partly through exosomal miRNAs. Nature. (2017) 548:52–7. doi: 10.1038/nature23282 PMC5999038 28746310

[84] Dailah HG. Potential of therapeutic small molecules in apoptosis regulation in the treatment of neurodegenerative diseases: an updated review. Molecules. (2022) 27(21):7207. doi: 10.3390/molecules27217207 36364033 PMC9654492

[85] Luo H Ye G Liu Y Huang D Luo Q Chen W. miR-150-3p enhances neuroprotective effects of neural stem cell exosomes after hypoxic-ischemic brain injury by targeting CASP2. Neurosci Lett. (2022) 779:136635. doi: 10.1016/j.neulet.2022.136635 35436510

[86] Liu Q Tan Y Qu T Zhang J Duan X Xu H. Therapeutic mechanism of human neural stem cell-derived extracellular vesicles against hypoxia-reperfusion injury in *vitro* . Life Sci. (2020) 254:117772. doi: 10.1016/j.lfs.2020.117772 32437794

[87] Galluzzi L Bravo-San Pedro JM Blomgren K Kroemer G. Autophagy in acute brain injury. Nat Rev Neurosci. (2016) 17:467–84. doi: 10.1038/nrn.2016.51 27256553

[88] Zhang HY Wang ZG Wu FZ Kong XX Yang J Lin BB. Regulation of autophagy and ubiquitinated protein accumulation by bFGF promotes functional recovery and neural protection in a rat model of spinal cord injury. Mol Neurobiol. (2013) 48:452–64. doi: 10.1007/s12035-013-8432-8 23516099

[89] Zhang L Han P. Neural stem cell-derived exosomes suppress neuronal cell apoptosis by activating autophagy via miR-374-5p/STK-4 axis in spinal cord injury. J Musculoskelet Neuronal Interact. (2022) 22(3):411–21. PMC9438516 36046998

[90] Liu B Teschemacher AG Kasparov S. Neuroprotective potential of astroglia. J Neurosci Res. (2017) 95:2126–39. doi: 10.1002/jnr.24140 28836687

[91] Lizarraga-Valderrama LR Sheridan GK. Extracellular vesicles and intercellular communication in the central nervous system. FEBS Lett. (2021) 595(10):1391–410. doi: 10.1002/1873-3468.14074 33728650

[92] Koutsaliaris IK Moschonas IC Pechlivani LM Tsouka AN Tselepis AD. Inflammation, oxidative stress, vascular aging and atherosclerotic ischemic stroke. Curr Med Chem. (2022) 29(34):5496–509. doi: 10.2174/0929867328666210921161711 34547993

[93] Li Z Zhao T Ding J Gu H Wang Q Wang Y. A reactive oxygen species-responsive hydrogel encapsulated with bone marrow derived stem cells promotes repair and regeneration of spinal cord injury. Bioact Mater. (2023) 19:550–68. doi: 10.1016/j.bioactmat.2022.04.029 PMC9108756 35600969

[94] Zhang R Mao W Niu L Bao W Wang Y Wang Y. NSC-derived exosomes enhance therapeutic effects of NSC transplantation on cerebral ischemia in mice. Elife. (2023) 12:e84493. doi: 10.7554/elife.84493 37104115 PMC10139690

[95] Sun X Jung JH Arvola O Santoso MR Giffard RG Yang PC. Stem cell-derived exosomes protect astrocyte cultures from *in vitro* ischemia and decrease injury as post-stroke intravenous therapy. Front Cell Neurosci. (2019) 13:394. doi: 10.3389/fncel.2019.00394 31551712 PMC6733914

[96] Hou BR Jiang C Wang ZN Ren HJ. Exosome-mediated crosstalk between microglia and neural stem cells in the repair of brain injury. Neural Regener Res. (2020) 15:1023–4. doi: 10.4103/1673-5374.270302 PMC7034280 31823874

[97] Morton MC Neckles VN Seluzicki CM Holmberg JC Feliciano DM. Neonatal subventricular zone neural stem cells release extracellular vesicles that act as a microglial morphogen. Cell Rep. (2018) 23:78–89. doi: 10.1016/j.celrep.2018.03.037 29617675

[98] Kumar M Sahu SK Kumar R Subuddhi A Maji RK Jana K. MicroRNA let-7 modulates the immune response to Mycobacterium tuberculosis infection via control of A20, an inhibitor of the NF-κB pathway. Cell Host Microbe. (2015) 17:345–56. doi: 10.1016/j.chom.2015.01.007 25683052

[99] Yao H Ma R Yang L Hu G Chen X Duan M. MiR-9 promotes microglial activation by targeting MCPIP1. Nat Commun. (2014) 5:4386. doi: 10.1038/ncomms5386 25019481 PMC4104446

[100] Lehmann SM Krüger C Park B Derkow K Rosenberger K Baumgart J. An unconventional role for miRNA: let-7 activates Toll-like receptor 7 and causes neurodegeneration. Nat Neurosci. (2012) 15:827–35. doi: 10.1038/nn.3113 22610069

[101] Zhang GY Wang J Jia YJ Han R Li P Zhu DN. MicroRNA-9 promotes the neuronal differentiation of rat bone marrow mesenchymal stem cells by activating autophagy. Neural Regener Res. (2015) 10:314–20. doi: 10.4103/1673-5374.143439 PMC4392682 25883633

[102] Li X Zhu Y Wang Y Xia X Zheng JC. Neural stem/progenitor cell-derived extracellular vesicles: A novel therapy for neurological diseases and beyond. MedComm (2020). (2023) 4:e214. doi: 10.1002/mco2.214 36776763 PMC9905070

[103] Smith SM Giedzinski E Angulo MC Lui T Lu C Park AL. Functional equivalence of stem cell and stem cell-derived extracellular vesicle transplantation to repair the irradiated brain. Stem Cells Transl Med. (2020) 9:93–105. doi: 10.1002/sctm.18-0227 31568685 PMC6954724

[104] Peng J Yu Z Xiao R Hu X Xia Y. Exosomal ZEB1 derived from neural stem cells reduces inflammation injury in OGD/R-treated microglia via the GPR30-TLR4-NF-κB axis. Neurochemical Res. (2023) 48(6):1811–21. doi: 10.1007/s11064-023-03866-3 36717511

[105] Webb RL Kaiser EE Jurgielewicz BJ Spellicy S Scoville SL Thompson TA. Human neural stem cell extracellular vesicles improve recovery in a porcine model of ischemic stroke. Stroke. (2018) 49:1248–56. doi: 10.1161/strokeaha.117.020353 PMC5916046 29650593

[106] Tian T Cao L He C Ye Q Liang R You W. Targeted delivery of neural progenitor cell-derived extracellular vesicles for anti-inflammation after cerebral ischemia. Theranostics. (2021) 11:6507–21. doi: 10.7150/thno.56367 PMC8120222 33995671

[107] Zheng X Zhang L Kuang Y Venkataramani V Jin F Hein K. Extracellular vesicles derived from neural progenitor cells—-a preclinical evaluation for stroke treatment in mice. Trans Stroke Res. (2021) 12:185–203. doi: 10.1007/s12975-020-00814-z PMC7803677 32361827

[108] Zhu ZH Jia F Ahmed W Zhang GL Wang H Lin CQ. Neural stem cell-derived exosome as a nano-sized carrier for BDNF delivery to a rat model of ischemic stroke. Neural Regener Res. (2023) 18:404–9. doi: 10.4103/1673-5374.346466 PMC9396474 35900437

[109] Apodaca LA Baddour AAD Garcia C Jr. Alikhani L Giedzinski E Ru N. Human neural stem cell-derived extracellular vesicles mitigate hallmarks of Alzheimer’s disease. Alzheimers Res Ther. (2021) 13:57. doi: 10.1186/s13195-021-00791-x 33676561 PMC7937214

[110] Li B Liu J Gu G Han X Zhang Q Zhang W. Impact of neural stem cell-derived extracellular vesicles on mitochondrial dysfunction, sirtuin 1 level, and synaptic deficits in Alzheimer’s disease. J Neurochem. (2020) 154:502–18. doi: 10.1111/jnc.15001 32145065

[111] Khan MI Jeong ES Khan MZ Shin JH Kim JD. Stem cells-derived exosomes alleviate neurodegeneration and Alzheimer’s pathogenesis by ameliorating neuroinflamation, and regulating the associated molecular pathways. Sci Rep. (2023) 13:15731. doi: 10.1038/s41598-023-42485-4 37735227 PMC10514272

[112] Rong Y Liu W Lv C Wang J Luo Y Jiang D. Neural stem cell small extracellular vesicle-based delivery of 14-3-3t reduces apoptosis and neuroinflammation following traumatic spinal cord injury by enhancing autophagy by targeting Beclin-1. Aging (Albany NY). (2019) 11:7723–45. doi: 10.18632/aging.102283 PMC6782003 31563124

[113] Ma K Xu H Zhang J Zhao F Liang H Sun H. Insulin-like growth factor-1 enhances neuroprotective effects of neural stem cell exosomes after spinal cord injury via an miR-219a-2-3p/YY1 mechanism. Aging (Albany NY). (2019) 11:12278–94. doi: 10.18632/aging.102568 PMC6949049 31848325

[114] Long Q Upadhya D Hattiangady B Kim DK An SY Shuai B. Intranasal MSC-derived A1-exosomes ease inflammation, and prevent abnormal neurogenesis and memory dysfunction after status epilepticus. Proc Natl Acad Sci U.S.A. (2017) 114:E3536–e3545. doi: 10.1073/pnas.1703920114 28396435 PMC5410779

[115] Xiong Y Mahmood A Chopp M. Emerging potential of exosomes for treatment of traumatic brain injury. Neural Regener Res. (2017) 12:19–22. doi: 10.4103/1673-5374.198966 PMC5319225 28250732

[116] Ahmadian S Jafari N Tamadon A Ghaffarzadeh A Rahbarghazi R Mahdipour M. Different storage and freezing protocols for extracellular vesicles: a systematic review. Stem Cell Res Ther. (2024) 15:453. doi: 10.1186/s13287-024-04005-7 39593194 PMC11600612

[117] Wu JY Li YJ Hu XB Huang S Xiang DX. Preservation of small extracellular vesicles for functional analysis and therapeutic applications: a comparative evaluation of storage conditions. Drug Delivery. (2021) 28:162–70. doi: 10.1080/10717544.2020.1869866 PMC7808382 33427518

[118] Campbell BCV Khatri P. Stroke. Lancet. (2020) 396(10244):129–42. doi: 10.1016/S0140-6736(20)31179-X 32653056

[119] Fugate JE Rabinstein AA. Update on intravenous recombinant tissue plasminogen activator for acute ischemic stroke. Mayo Clin Proc. (2014) 89:960–72. doi: 10.1016/j.mayocp.2014.03.001 24775222

[120] Gao M Yao H Dong Q Zhang Y Yang Y Zhang Y. Neurotrophy and immunomodulation of induced neural stem cell grafts in a mouse model of closed head injury. Stem Cell Res. (2017) 23:132–42. doi: 10.1016/j.scr.2017.07.015 28743043

[121] Liang Y Duan L Lu J Xia J. Engineering exosomes for targeted drug delivery. Theranostics. (2021) 11:3183–95. doi: 10.7150/thno.52570 PMC7847680 33537081

[122] Scheltens P Blennow K Breteler MM De Strooper B Frisoni GB Salloway S. Alzheimer’s disease. Lancet. (2016) 388(10043):505–17. doi: 10.1016/S0140-6736(15)01124-1 26921134

[123] Long JM Holtzman DM. Alzheimer disease: an update on pathobiology and treatment strategies. Cell. (2019) 179:312–39. doi: 10.1016/j.cell.2019.09.001 PMC6778042 31564456

[124] Spangenberg EE Lee RJ Najafi AR Rice RA Elmore MR Blurton-Jones M. Eliminating microglia in Alzheimer’s mice prevents neuronal loss without modulating amyloid-β pathology. Brain. (2016) 139:1265–81. doi: 10.1093/brain/aww016 PMC5006229 26921617

[125] Elahi FM Casaletto KB La Joie R Walters SM Harvey D Wolf A. Plasma biomarkers of astrocytic and neuronal dysfunction in early- and late-onset Alzheimer’s disease. Alzheimers Dement. (2020) 16:681–95. doi: 10.1016/j.jalz.2019.09.004 PMC7138729 31879236

[126] Chen M Liu J Wu W Guo T Yuan J Wu Z. SIRT1 restores mitochondrial structure and function in rats by activating SIRT3 after cerebral ischemia/reperfusion injury. Cell Biol Toxicol. (2024) 40:31. doi: 10.1007/s10565-024-09869-2 38767771 PMC11106166

[127] Jiang B Shen RF Bi J Tian XS Hinchliffe T Xia Y. Catalpol: A potential therapeutic for neurodegenerative diseases. Curr Medicinal Chem. (2015) 22:1278–91. doi: 10.2174/0929867322666150114151720 25620103

[128] Poewe W Seppi K Tanner CM Halliday GM Brundin P Volkmann J. Parkinson disease. Nat Rev Dis Primers. (2017) 3:17013. doi: 10.1038/nrdp.2017.13 28332488

[129] Yu H Sun T An J Wen L Liu F Bu Z. Potential roles of exosomes in parkinson’s disease: from pathogenesis, diagnosis, and treatment to prognosis. Front Cell Dev Biol. (2020) 8:86. doi: 10.3389/fcell.2020.00086 32154247 PMC7047039

[130] Trist BG Hare DJ Double KL. Oxidative stress in the aging substantia nigra and the etiology of Parkinson’s disease. Aging Cell. (2019) 18:e13031. doi: 10.1111/acel.13031 31432604 PMC6826160

[131] Reeve AK Grady JP Cosgrave EM Bennison E Chen C Hepplewhite PD. Mitochondrial dysfunction within the synapses of substantia nigra neurons in Parkinson’s disease. NPJ Parkinsons Dis. (2018) 4:9. doi: 10.1038/s41531-018-0044-6 29872690 PMC5979968

[132] Roser AE Caldi Gomes L Halder R Jain G Maass F Tönges L. miR-182-5p and miR-183-5p act as GDNF mimics in dopaminergic midbrain neurons. Mol Ther Nucleic Acids. (2018) 11:9–22. doi: 10.1016/j.omtn.2018.01.005 29858093 PMC5849806

[133] Chen M Lin Y Guo W Chen L. BMSC-Derived Exosomes Carrying miR-26a-5p Ameliorate Spinal Cord Injury via Negatively Regulating EZH2 and Activating the BDNF-TrkB-CREB Signaling. Mol Neurobiol. (2024) 61:8156–74. doi: 10.1007/s12035-024-04082-y 38478142

[134] Van Den Berg ME Castellote JM Mahillo-Fernandez I De Pedro-Cuesta J. Incidence of spinal cord injury worldwide: a systematic review. Neuroepidemiology. (2010) 34:184–92;discussion 192. doi: 10.1159/000279335 20130419

[135] Jain NB Ayers GD Peterson EN Harris MB Morse L O’connor KC. Traumatic spinal cord injury in the United States 1993-2012. Jama. (2015) 313:2236–43. doi: 10.1001/jama.2015.6250 PMC4712685 26057284

[136] Li S Dinh HTP Matsuyama Y Sato K Yamagishi S. Molecular mechanisms in the vascular and nervous systems following traumatic spinal cord injury. Life (Basel). (2022) 13(1):9. doi: 10.3390/life13010009 36675958 PMC9866624

[137] Holmes D. Spinal-cord injury: spurring regrowth. Nature. (2017) 552:S49. doi: 10.1038/d41586-017-07550-9 29239374

[138] Sim-Selley LJ Wilkerson JL Burston JJ Hauser KF Mclane V Welch SP. Differential tolerance to FTY720-induced antinociception in acute thermal and nerve injury mouse pain models: role of sphingosine-1-phosphate receptor adaptation. J Pharmacol Exp Ther. (2018) 366:509–18. doi: 10.1124/jpet.118.248260 PMC6090176 29945931

[139] Mas-Bargues C Borrás C. Importance of stem cell culture conditions for their derived extracellular vesicles therapeutic effect. Free Radic Biol Med. (2021) 168:16–24. doi: 10.1016/j.freeradbiomed.2021.03.028 33781893

[140] Sims B Gu L Krendelchtchikov A Matthews QL. Neural stem cell-derived exosomes mediate viral entry. Int J Nanomedicine. (2014) 9:4893–7. doi: 10.2147/IJN.S70999 PMC4211904 25364247

[141] Marchetti B Tirolo C L’episcopo F Caniglia S Testa N Smith JA. Parkinson’s disease, aging and adult neurogenesis: Wnt/β-catenin signalling as the key to unlock the mystery of endogenous brain repair. Aging Cell. (2020) 19:e13101. doi: 10.1111/acel.13101 32050297 PMC7059166

[142] Xu M Feng T Liu B Qiu F Xu Y Zhao Y. Engineered exosomes: desirable target-tracking characteristics for cerebrovascular and neurodegenerative disease therapies. Theranostics. (2021) 11:8926–44. doi: 10.7150/thno.62330 PMC8419041 34522219

[143] Jin S Lv Z Kang L Wang J Tan C Shen L. Next generation of neurological therapeutics: Native and bioengineered extracellular vesicles derived from stem cells. Asian J Pharm Sci. (2022) 17:779–97. doi: 10.1016/j.ajps.2022.10.002 PMC9800941 36600903

[144] Nalamolu KR Venkatesh I Mohandass A Klopfenstein JD Pinson DM Wang DZ. Exosomes secreted by the cocultures of normal and oxygen-glucose-deprived stem cells improve post-stroke outcome. Neuromolecular Med. (2019) 21:529–39. doi: 10.1007/s12017-019-08540-y PMC7272187 31077035

[145] Chen C Chang ZH Yao B Liu XY Zhang XW Liang J. 3D printing of interferon γ-preconditioned NSC-derived exosomes/collagen/chitosan biological scaffolds for neurological recovery after TBI. Bioact Mater. (2024) 39:375–91. doi: 10.1016/j.bioactmat.2024.05.026 PMC11153920 38846528

[146] Liang Y Iqbal Z Lu J Wang J Zhang H Chen X. Cell-derived nanovesicle-mediated drug delivery to the brain: Principles and strategies for vesicle engineering. Mol Ther. (2022) 31(5):1207–24. doi: 10.1016/j.ymthe.2022.10.008 PMC10188644 36245129

[147] Qian C Wang Y Ji Y Chen D Wang C Zhang G. Neural stem cell−derived exosomes transfer miR−124−3p into cells to inhibit glioma growth by targeting FLOT2. Int J Oncol. (2022) 61(4):115. doi: 10.3892/ijo.2022.5405 35929514 PMC9387557

[148] Wiklander OP Nordin JZ O’loughlin A Gustafsson Y Corso G Mäger I. Extracellular vesicle *in vivo* biodistribution is determined by cell source, route of administration and targeting. J Extracell Vesicles. (2015) 4:26316. doi: 10.3402/jev.v4.26316 25899407 PMC4405624

[149] Kumar P Wu H Mcbride JL Jung KE Kim MH Davidson BL. Transvascular delivery of small interfering RNA to the central nervous system. Nature. (2007) 448:39–43. doi: 10.1038/nature05901 17572664

[150] Salunkhe S Dheeraj Basak M Chitkara D Mittal A. Surface functionalization of exosomes for target-specific delivery and *in vivo* imaging & tracking: Strategies and significance. J Control Release. (2020) 326:599–614. doi: 10.1016/j.jconrel.2020.07.042 32730952

[151] Alvarez-Erviti L Seow Y Yin H Betts C Lakhal S Wood MJ. Delivery of siRNA to the mouse brain by systemic injection of targeted exosomes. Nat Biotechnol. (2011) 29:341–5. doi: 10.1038/nbt.1807 21423189

[152] Yang J Zhang X Chen X Wang L Yang G. Exosome Mediated Delivery of miR-124 Promotes Neurogenesis after Ischemia. Mol Ther Nucleic Acids. (2017) 7:278–87. doi: 10.1016/j.omtn.2017.04.010 PMC5415550 28624203

[153] Du S Guan Y Xie A Yan Z Gao S Li W. Extracellular vesicles: a rising star for therapeutics and drug delivery. J Nanobiotechnology. (2023) 21:231. doi: 10.1186/s12951-023-01973-5 37475025 PMC10360328

[154] Noren Hooten N Yáñez-Mó M Derita R Russell A Quesenberry P Ramratnam B. Hitting the Bullseye: Are extracellular vesicles on target? J Extracell Vesicles. (2020) 10:e12032. doi: 10.1002/jev2.12032 33708359 PMC7890543

[155] Lee ES Ko H Kim CH Kim HC Choi SK Jeong SW. Disease-microenvironment modulation by bare- or engineered-exosome for rheumatoid arthritis treatment. Biomater Res. (2023) 27:81. doi: 10.1186/s40824-023-00418-2 37635253 PMC10464174

